# A Cluster-Guided Screening Framework for Prioritizing Natural Product Candidates Against HIV-1 from the KNApSAcK Database

**DOI:** 10.3390/ph19101534

**Published:** 2026-09-27

**Authors:** Muhammad Alqaaf, Md. Abdullah Al Mamun, Ahmad Kamal Nasution, A S M Nazrul Islam, Retno Supriyanti, Naoaki Ono, Shigehiko Kanaya, Md. Altaf-Ul-Amin

**Affiliations:** 1 Graduate School of Science and Technology, Nara Institute of Science and Technology, Ikoma 630-0192, Nara, Japan; muhammad.alqaaf_subandoko.mb5@naist.ac.jp (M.A.); kamal.nasution@naist.ac.jp (A.K.N.); a.s_m_nazrul_islam.aw1@naist.ac.jp (A.S.M.N.I.); nono@is.naist.jp (N.O.); skanaya@is.naist.jp (S.K.); 2 Faculty of Mechanical, Electrical, and Electronics Engineering, Shimane University, Matsue 690-8504, Shimane, Japan; n23d104@matsu.shimane-u.ac.jp; 3 Department of Electrical Engineering, Jenderal Soedirman University, Purbalingga 53371, Central Java, Indonesia; retno_supriyanti@unsoed.ac.id; 4 Faculty of Information and Communication Engineering, Osaka Electro-Communication University, Neyagawa 572-8530, Osaka, Japan

**Keywords:** HIV-1, natural products, secondary metabolites, KNApSAcK, virtual screening, molecular docking, sequence-based clustering, ADME prediction

## Abstract

**Background:** Plant-derived natural products are a productive antiviral scaffold source, yet secondary metabolite libraries remain underexplored against HIV-1 amid extensive target-structure redundancy. This study presents a cluster-guided framework for HIV-1 inhibitor prioritization from the KNApSAcK database. **Methods:** A total of 64,166 SMs were converted to SMILES and queried against BindingDB to identify reported HIV-1 integrase, protease, and reverse-transcriptase associations. A total of 295 HIV-1 protein sequences were aligned and partitioned using DPClusSBO; representative structures (6VDK, 1MUI, 6ELI) per cluster were docked against cluster-mapped SMs using SMINA. Prioritized SMs were evaluated with SwissADME and benchmarked against ChEMBL HIV-1 inhibitors. **Results:** Clustering resolved three non-overlapping groups (integrase, *n* = 135; protease, *n* = 117; reverse transcriptase, *n* = 42), mapping 285, 124, and 410 SMs, respectively. Predicted docking scores ranged from −18.91 to −4.72 kcal/mol (integrase), −26.54 to −7.87 kcal/mol (protease), and −26.83 to −4.59 kcal/mol (reverse transcriptase). ADME-prioritized reverse-transcriptase compounds scored more favorably than the matched NNRTI reference set (*p* < 0.001), requiring experimental confirmation of binding affinity. DUD-E enrichment validation showed strong discriminative validity for integrase and protease (ROC-AUC 0.85, 0.78) but not reverse transcriptase (ROC-AUC 0.53), consistent with weaker pose reproduction for the latter. **Conclusions:** The framework reduced target redundancy and computationally prioritized natural product candidates for HIV-1 as hypothesis-generating predictions requiring experimental validation.

## 1. Introduction

Human immunodeficiency virus type 1 (HIV-1) remains a significant global public health challenge despite notable advancements in antiretroviral therapy (ART), with tens of millions of individuals currently living with the infection worldwide, and over one million new infections reported annually [1]. The extensive implementation of combination ART has markedly reduced HIV-related morbidity and mortality, effectively transforming HIV-1 infection into a manageable chronic condition for many patients [2]. However, the long-term efficacy of treatment is compromised by the persistent emergence of drug-resistant viral strains, which limits therapeutic options and necessitates frequent regimen modifications [3,4]. The exceptionally high replication rate of HIV-1, coupled with its pronounced genetic variability, facilitates the rapid accumulation of resistance-associated mutations in key viral enzymes, such as reverse transcriptase, protease, and integrase, even under sustained drug pressure [5,6]. Consequently, the ongoing identification of novel antiviral compounds with distinct chemical scaffolds and alternative binding mechanisms remains a critical priority in HIV-1 drug discovery efforts [2].

HIV-1 replication depends on the sequential, non-redundant action of the three enzymes examined in this study, each a validated and clinically exploited antiretroviral target. Following viral entry, reverse transcriptase (RT), a p66/p51 heterodimer, converts the single-stranded viral RNA genome into double-stranded proviral DNA through its RNA/DNA-dependent DNA polymerase activity, using a canonical two-metal-ion mechanism centered on a catalytic aspartate triad (Asp110, Asp185, Asp186), while its separate RNase H domain degrades the RNA template of the resulting RNA/DNA hybrid [2,5]. Non-nucleoside reverse-transcriptase inhibitors (NNRTIs) do not compete with substrate at this polymerase active site; instead, they bind an allosteric hydrophobic pocket approximately 10 Å away, formed principally by aromatic residues (including Tyr181, Tyr188, and Trp229) from the p66 subunit, and inhibit polymerization by distorting the geometry of the adjacent catalytic triad rather than by direct active-site competition [7]. The resulting proviral DNA is then integrated into the host chromosome by integrase, which catalyzes two sequential magnesium-dependent reactions, 3’-processing and strand transfer, through a DDE catalytic triad (Asp64, Asp116, Glu152) that coordinates the two catalytic Mg^2+^ ions targeted by integrase strand-transfer inhibitors (INSTIs) such as dolutegravir [8,9]. Finally, during viral particle maturation, the homodimeric aspartic protease cleaves the Gag and Gag-Pol polyproteins at multiple specific sites; catalysis proceeds via a water molecule activated by two catalytic aspartates (Asp25/Asp25′ contributed one from each monomer at the dimer interface), with a flexible flap region (residues approximately 43–58, including Ile50) that opens and closes over the substrate-binding cleft to regulate access to the active site [10,11]. Because these enzymes act at distinct stages of the viral life cycle through mechanistically unrelated chemistries, they remain a preferred focus for combination antiretroviral therapy and, correspondingly, for the natural product screening pursued in this study.

Computational virtual screening, particularly structure-based docking, has emerged as a pivotal element in contemporary antiviral drug discovery, as it facilitates the rapid prioritization of candidate ligands before experimental validation [12,13,14]. Nevertheless, numerous in silico studies targeting HIV-1 are constrained not by the docking process itself but by the selection, scaling, and organization of compound libraries and protein targets prior to docking [3,15,16,17]. On the target side, HIV-1 enzymes, such as protease, reverse transcriptase, and integrase, are represented by numerous experimentally determined structures across various constructs, ligand-bound states, and resistance-associated variants. These variations can modify the binding-site geometry and interactions, thereby significantly influencing the docking outcomes based on the selected target structures [2,3,15]. A practical challenge arises when docking is conducted across multiple highly similar target structures without a systematic organizational strategy, leading to redundant screenings that are computationally intensive and difficult to interpret. Near-duplicate targets can produce highly correlated docking scores and rankings, potentially biasing prioritization and inflating the perceived number of “hits” while incurring substantial computational costs. Consequently, a target-organization step that consolidates redundant HIV-1 protein variants while preserving biologically meaningful diversity can enhance the scalability and interpretability of large-scale screening campaigns.

Natural products continue to serve as invaluable sources of bioactive chemical scaffolds, significantly contributing to contemporary drug discovery [13,15,18,19]. Secondary metabolites (SMs) derived from plants and other natural sources exhibit remarkable structural diversity, stereochemical complexity, and a rich variation of functional groups, which are seldom replicated by synthetic compound libraries alone [13,18,19]. Despite this established potential, extensive natural product repositories remain underutilized in HIV-1 virtual screening, with numerous studies focusing on limited subsets rather than entire database collections [15,20]. Comprehensive resources, such as the KNApSAcK database, which curates tens of thousands of plant-derived SMs with chemical structures, present an opportunity for extensive exploration [21,22]. However, the scale and heterogeneity of these datasets pose practical challenges for their integration into conventional docking workflows. Furthermore, linking natural metabolites to relevant HIV-1 targets is enhanced by integrating external evidence sources such as BindingDB, which aggregates experimentally reported protein–ligand binding data and facilitates systematic mapping between small molecules and targets [13,23,24].

In this study, we addressed two key challenges in HIV-1 virtual screening: (i) target redundancy arising from the large number of structurally similar viral protein variants and (ii) the complexity associated with large-scale natural product libraries. To overcome these limitations, we propose a cluster-guided screening framework aligned with a scalable computational pipeline. This design deliberately inverts the target-to-compound screening direction previously validated for SARS-CoV-2, in which a single viral spike protein sequence was queried against BindingDB to retrieve candidate binding molecules and, from these, prioritize small-molecule inhibitors [13]; here, the search instead begins from a large compound library and uses BindingDB associations to identify the relevant protein targets. Demonstrating that the same sequence-similarity-network-guided, BindingDB-mediated strategy generalizes to this reverse, compound-first direction is itself a methodological contribution of this study, independent of any specific HIV-1 candidate it prioritizes. Plant-derived secondary metabolites were retrieved from KNApSAcK [21,22] and mapped to HIV-1-relevant targets using BindingDB [23,24], followed by the collection of corresponding protein sequences and structures from the Protein Data Bank [25]. A protein similarity network was then constructed based on pairwise sequence identity (≥50%) and clustered using DPClusSBO to group closely related HIV-1 protein variants [13,26,27,28]. This sequence-based clustering provides a principled and scalable approach to reduce redundancy while preserving the representative structural and functional diversity [29]; because integrase, protease, and reverse transcriptase are already known to be structurally and mechanistically unrelated, the clustering is expected to recover exactly these three established enzyme classes rather than reveal novel functional groupings, and its value lies instead in reducing the 295 individual HIV-1 protein chains considered here to three representative structures within these known class boundaries. Representative structures from each cluster were subsequently used for high-throughput docking with SMINA [30,31], with binding pockets identified via fpocket [32], and candidate compounds were prioritized using SwissADME-based pharmacokinetic profiling [33]. Rather than evaluating therapeutic efficacy, this study focused on establishing a scalable and biologically informed computational framework for candidate prioritization. Accordingly, the results are presented as computational hypotheses intended to guide experimental validation and downstream optimizations. By integrating whole-database metabolite screening with sequence-similarity-based target organization and systematic post-docking filtering, this approach enhances scalability, reduces redundancy-driven bias, and improves the interpretability of large-scale virtual screening, while providing a transferable strategy for redundancy-aware analysis in structurally heterogeneous target systems.

## 2. Results

### 2.1. Sequence-Based Clustering of HIV-1 Protein Structures

**Figure 1:**
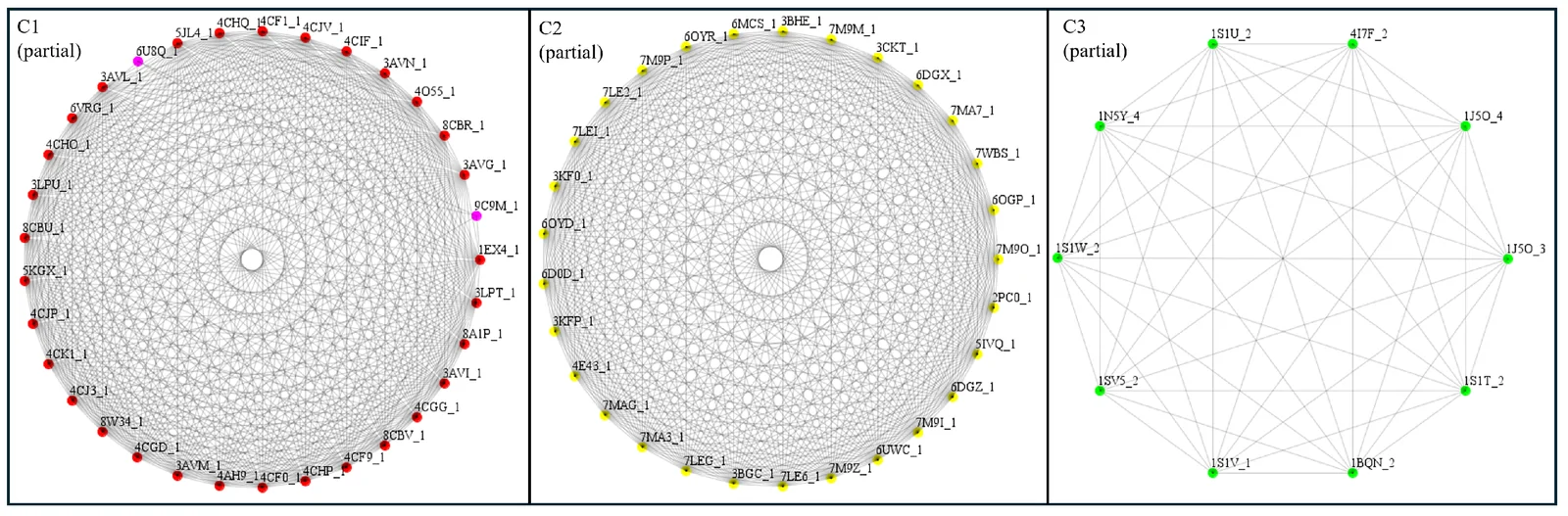
Sampled network visualization of the structural clustering of HIV-1 enzymes. The data were reduced for better data visualization.

All-versus-all pairwise sequence alignment of 295 HIV-1 protein chains generated 43,365 pairwise comparisons. A 50% sequence identity threshold was applied to retain high-confidence similarity relationships for the network construction. DPClusSBO visualizes the clustering results as network representations, with nodes representing protein structures and edges representing structural similarities (Figure 1).

DPClusSBO clustering successfully identified three distinct clusters consisting of three types of HIV proteins, with cluster sizes ranging from 42 to 135 members (Table 1; Supplementary Materials S2, Table S8). Cluster C1 comprised HIV-1 integrase chains, Cluster C2 HIV-1 protease chains, and Cluster C3 HIV-1 reverse-transcriptase chains. Because the clustering operated solely on pairwise sequence identity, these groups reflect sequence-level relationships among the retrieved chains; we do not infer conformational or dynamic properties from the clustering itself. The images of each cluster in Figure 1 are only partially shown for ease of visualization. Complete images of the clusters are shown in Figure S1 (Supplementary Material S2).

This three-way split reproduces the already-established functional classification of these enzymes: integrase, protease, and reverse transcriptase are structurally and mechanistically unrelated proteins, so recovering exactly these three groups from sequence similarity alone is an expected consistency check on the clustering procedure rather than a novel finding in itself. The substantive contribution of this step is not the discovery of new functional groupings but the reduction from 295 individual HIV-1 protein chains to three representative structures within these already-known class boundaries, which is what makes redundancy-aware docking of the full KNApSAcK library computationally tractable (Section 1).

Structures representing Clusters 1 and 2 were selected based on earlier studies [34,35,36,37], whereas the representative structure for Cluster 3 was selected based on its high crystallographic resolution of 2.2 Å, its representation as a complete p66/p51 heterodimer, and its co-crystallization with rilpivirine, an FDA-approved non-nucleoside reverse-transcriptase inhibitor (NNRTI), within the NNRTI-binding pocket [38]. The representative structures selected for molecular docking were 6VDK for integrase, 1MUI for protease, and 6ELI for reverse transcriptase.

### 2.2. Distribution of Secondary Metabolites Across Protein Clusters

The distribution of SMs across the HIV-1 protein clusters based on the BindingDB search results is presented in Table 2. Clusters C1 (Integrases), C2 (Proteases), and C3 (Reverse Transcriptases) were associated with 285, 124, and 410 unique metabolites, respectively. This separation of metabolites across enzyme classes reflects the BindingDB associations carried through the clustering and filtering steps, and preserves the class assignments derived from that evidence. A complete list of SMs assigned to each cluster is provided in Supplementary Materials S1, Table S6.

**Figure 2:**
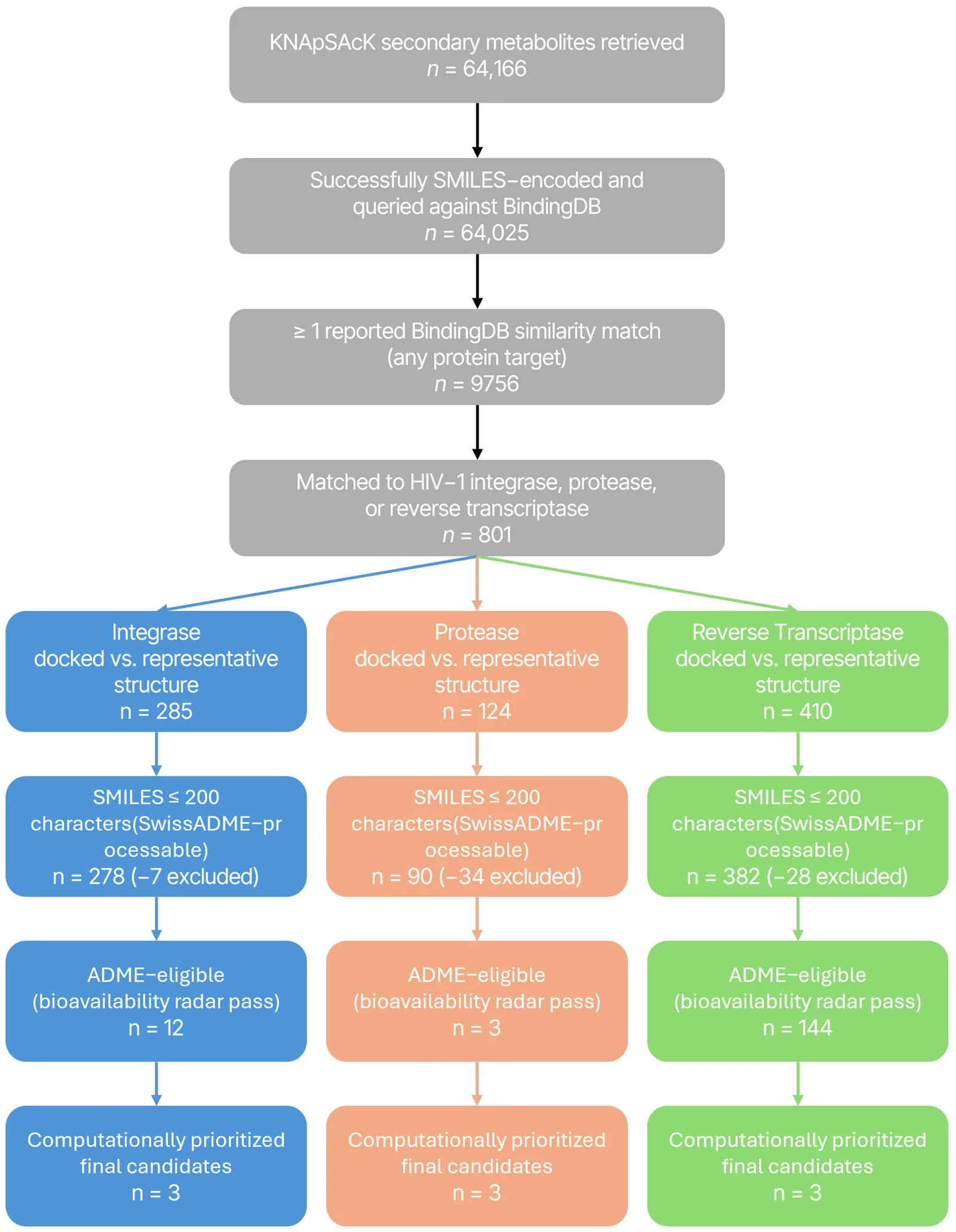
Compound attrition funnel from the initial KNApSAcK library to the final computationally prioritized candidates. Of the 64,166 secondary metabolites retrieved, 64,025 were successfully SMILES-encoded and queried against BindingDB; 9756 returned at least one reported similarity match to any protein target, of which 801 unique compounds mapped specifically to HIV-1 integrase, protease, or reverse transcriptase (285 + 124 + 410 = 819 cluster-compound assignments; 18 compounds were associated with more than one enzyme class—16 of these span the integrase/reverse-transcriptase pair alone—and were therefore docked against more than one representative structure). These were distributed across the three clusters (285, 124, and 410 compounds, respectively; Table 2), docked against each cluster’s representative structure, filtered by SwissADME bioavailability-radar eligibility (12, 3, and 144 compounds, respectively), and narrowed to three ADME-prioritized final candidates per cluster (nine in total).

Figure 2 summarizes the full compound-attrition pathway from the initial KNApSAcK library to the final prioritized candidates, tracing each filtering stage back to the underlying dataset counts rather than presenting only the endpoint numbers.

### 2.3. Docking Protocol Validation: Results

To assess whether the SMINA-based docking protocol could reproduce experimentally observed binding poses, each representative structure’s own co-crystallized native ligand was extracted from the original (un-stripped) coordinate file and redocked into its own binding site using the identical SMINA parameters as the main screening (Section 4.4). The three native ligands were dolutegravir (PDB ligand ID DLU) for 6VDK, lopinavir (AB1) for 1MUI, and rilpivirine (T27) for 6ELI. Pose accuracy was quantified as the heavy-atom root-mean-square deviation (RMSD) between the top-scoring redocked pose and the crystallographic ligand coordinates, using a symmetry-corrected structural alignment (OpenBabel 3.2.0 OBAlign). A grid-placement diagnostic additionally compared the native-ligand-centered grid against the fpocket-derived grid actually used for the main screening of each target (Table 3).

None of the three targets reproduced the crystallographic pose within the conventional ≤2 Å redocking-success threshold. The grid-placement diagnostic distinguished two distinct failure modes. For 1MUI and 6ELI, the fpocket-derived grid used for the main screening coincided closely with the native ligand site (1.10 Å and 3.19 Å, respectively), yet the top-scoring redocked pose still deviated substantially from the crystal structure. To determine whether this reflected insufficient conformational sampling, redocking was repeated at a higher exhaustiveness setting (=16, twice the default used throughout this study); this yielded only a marginal improvement for 1MUI (5.91 to 5.81 Å) and a modest improvement for 6ELI (5.61 to 4.98 Å), neither approaching the 2 Å threshold. As a second, independent correction attempt targeting the specific residues each pocket’s induced-fit behavior is attributed with this, rather than sampling depth generally, redocking was additionally repeated with flexible side chains, using SMINA’s native –flexres mode at the default exhaustiveness, at ILE50 of both protease monomers for 1MUI and at TYR181, TYR188, and TRP229 of the NNRTI pocket for 6ELI. This again yielded only a marginal improvement for 1MUI (5.91 to 5.82 Å) and a comparably modest improvement for 6ELI (5.61 to 4.97 Å), closely matching rather than superseding the exhaustiveness result above. Because neither increasing exhaustiveness fourfold (an exhaustiveness =32 run was also attempted but did not complete within a practical time budget for a single ligand, making a full-library redocking at that setting computationally infeasible) nor allowing these specific induced-fit residues to move meaningfully improved pose accuracy, the residual deviation is attributed to the size and conformational flexibility of these ligands (lopinavir is a large peptidomimetic; rilpivirine occupies an induced-fit non-nucleoside reverse-transcriptase inhibitor (NNRTI) pocket not fully accessible from a single static receptor conformation) rather than to inadequate sampling depth or an untried, fixed set of side-chain conformations, and is reported here as an inherent limitation of rigid-receptor empirical-scoring docking for these ligand classes rather than a correctable protocol error.

For 6VDK, the failure was more severe and mechanistically distinct: the native-ligand-centered grid was 28.78 Å away from the pocket originally selected for the integrase screening by the automated fpocket step. Inspection of the raw crystallographic structure showed that, in addition to four Mg^2+^ ions, the intasome coordinate file (PDB: 6VDK) contains two short viral DNA strands (chains E–H) that, together with the protein and metal ions, form the active site to which integrase strand-transfer inhibitors (INSTIs) such as dolutegravir bind. Because the receptor-preparation step (Section 4.4) retains only protein chains, the resulting protein-only structure lacks the DNA component of the true binding site, and fpocket consequently identified a different, DNA-independent cavity as the top-scoring pocket. This is a more fundamental limitation than the absence of explicit metal-ion scoring alone, so, unlike 1MUI and 6ELI, this was treated as a correctable protocol error rather than an inherent modeling limitation: the grid was re-centered directly on the native dolutegravir coordinates, and the entire cluster-1 compound library was subsequently redocked against this corrected grid (Section 4.4). All integrase docking results reported elsewhere in this study reflect this corrected protocol, not the original 28.78 Å-displaced grid diagnosed here (see also the footnote to Table 3). Even with this correction, the receptor itself still lacks the viral DNA strands that, together with the protein and Mg^2+^ ions, form the complete intasome active site. Re-centering the grid on the native ligand’s coordinates fixes where the search box is placed, not what is structurally present around it, so integrase-specific results should be interpreted with somewhat greater caution than the protease and reverse-transcriptase results, both for this structural incompleteness and because the corrected grid was chosen directly from the native ligand rather than validated by an independent pose-reproduction success (Table 3 shows that the redocked pose still misses the crystallographic pose by 6.44 Å).

All KNApSAcK candidates and FDA-approved/clinical reference compounds were nonetheless docked under an identical protocol per target, so relative rankings within each target remain internally consistent for compound prioritization purposes; however, this validation indicates that the absolute docking poses and docking scores reported here, particularly for integrase, should not be interpreted as accurate representations of the true binding mode.

**Figure 3:**
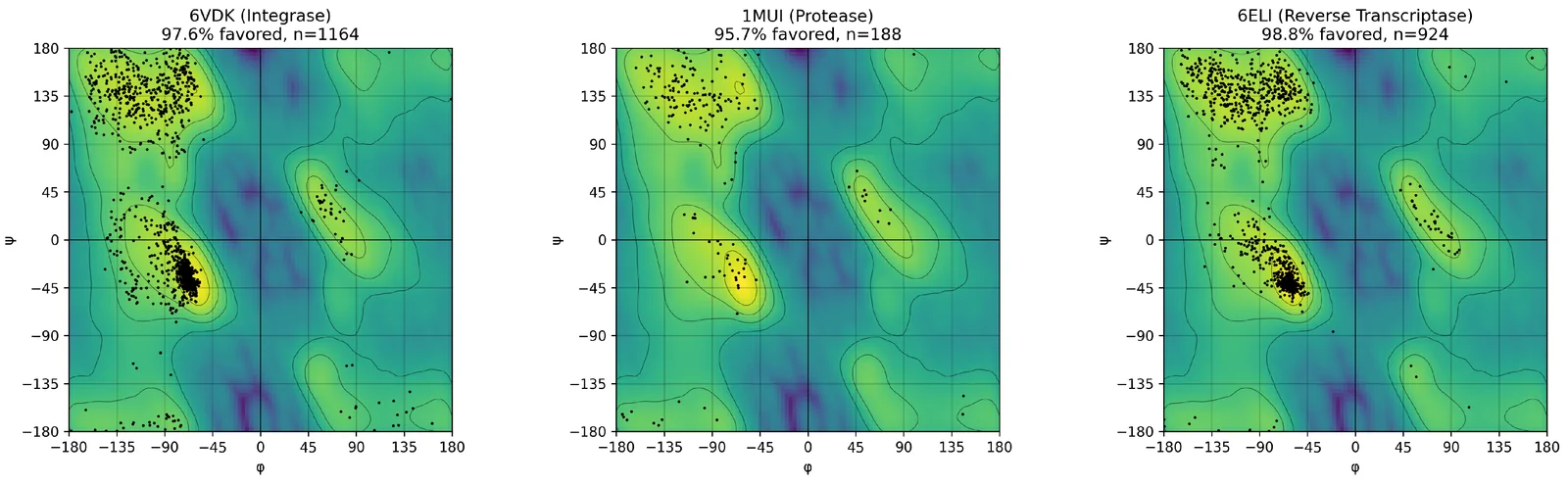
Ramachandran plots of the three representative structures (6VDK, 1MUI, 6ELI), showing backbone phi/psi dihedral angles (black points) against a reference density background derived from the Top8000 high-resolution structure dataset, where warmer colors (yellow) indicate the most densely populated, energetically favored conformational regions and cooler colors (blue) indicate rarely observed, energetically disallowed regions. Favored-region percentages are computed from the same density grid used for the contour shading.

As a complementary structural-quality check, backbone dihedral (phi/psi) angles for all three representative structures were computed with Biopython [39] and evaluated with a Ramachandran plot referenced against the Top8000 curated high-resolution structure dataset [40] (Figure 3). Residues fell within the favored region (density threshold enclosing 98% of the reference dataset’s probability mass) in 97.6% of 6VDK (1164 residues), 95.7% of 1MUI (188 residues), and 98.8% of 6ELI (924 residues), indicating that backbone geometry for all three representative structures is within the range expected for well-refined, good-quality structures and is not itself a source of concern for the docking results.

### 2.4. Retrospective DUD-E Enrichment Validation: Results

The validation above establishes that pose accuracy is limited for all three representative structures. This does not by itself indicate whether the docking score can still distinguish true binders from non-binders; a scoring function can, in principle, rank compounds informatively, even when the specific 3D pose it selects is not the crystallographic one. This was tested directly using the DUD-E enrichment protocol described in Section 4.6, with results summarized in Table 4.

**Figure 4:**
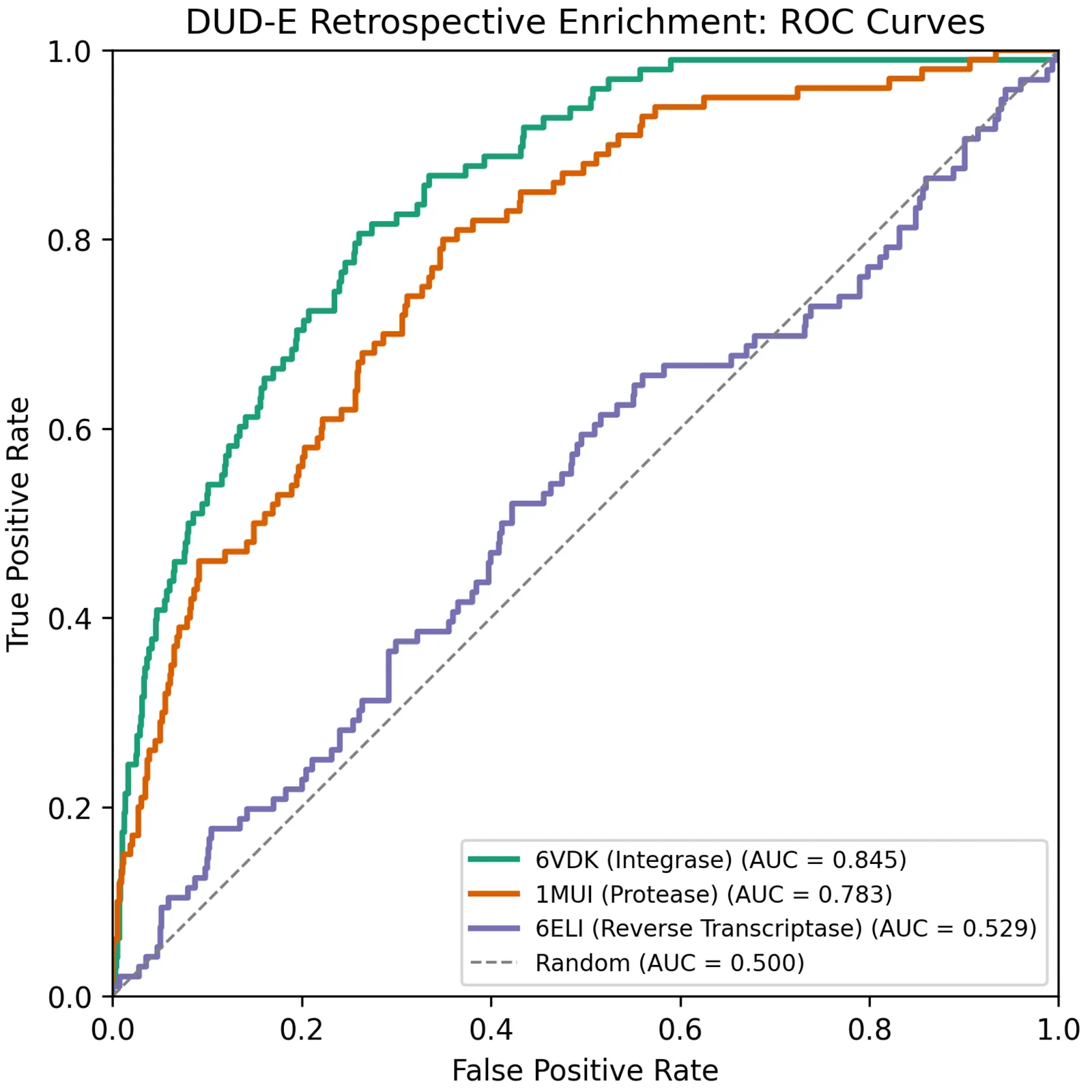
Receiver operating characteristic (ROC) curves for the retrospective DUD-E enrichment analysis (Table 4), plotting the true-positive rate (correctly ranked actives) against the false-positive rate (incorrectly ranked decoys) as the docking-score threshold is varied, for each of the three representative structures. The diagonal dashed line indicates the performance expected from a random, uninformative ranking (AUC = 0.500).

For integrase and protease, the docking score showed clear discriminative validity: ROC-AUC of 0.845 and 0.783, respectively, well above the 0.5 baseline expected of random ranking, with both curves rising well above the diagonal across the full threshold range (Figure 4), and enrichment factors of approximately 4- to 6-fold at the top 1–10% of the ranked list for both targets. This indicates that, despite the pose-accuracy limitations documented above, the relative ranking produced by this docking protocol for these two targets carries genuine information about which compounds are more likely to be true binders, rather than being internally consistent but otherwise uninformative noise; this directly addresses whether the docking-score-based prioritization used throughout this study has any real discriminative basis, complementing the internal-consistency argument made in Section 2.3.

For reverse transcriptase, by contrast, the docking score showed essentially no discriminative validity (ROC-AUC = 0.529, indistinguishable from random ranking, with the ROC curve tracking close to the diagonal throughout, as shown in Figure 4; enrichment factors at 5% and 10% close to 1). We do not consider this an unexplained anomaly: 6ELI already showed the least reliable pose reproduction of the three targets in this study (Section 2.3), attributed there to rilpivirine occupying an induced-fit NNRTI pocket not fully accessible from a single static receptor conformation. A weak enrichment result is consistent with that same underlying limitation, since a rigid receptor unable to capture the conformational adaptation this pocket undergoes on ligand binding would be expected to fail at both tasks together. We note, however, that protease shows a broadly similar pose-reproduction shortfall yet retains strong enrichment, so pose inaccuracy alone does not fully explain the reverse-transcriptase result; the NNRTI pocket’s particular degree of conformational plasticity, properties of the specific decoy set for this target, or a combination of factors may also contribute, and we report this as an honest limitation rather than resolve it further here. Docking-score-based prioritization for the reverse-transcriptase cluster should accordingly be read with more caution than for integrase or protease, consistent with the hedged, hypothesis-generating framing already applied to all candidate-level claims in this study (Section 3.2 et seq.).

### 2.5. Molecular Docking Performance Analysis

#### 2.5.1. Docking Score Distribution Across Clusters

The docking performance of SMs across HIV-1 protein clusters was evaluated by analyzing the distribution of the predicted docking scores obtained using SMINA (Supplementary Material S3, Tables S12–S14).

**Figure 5:**
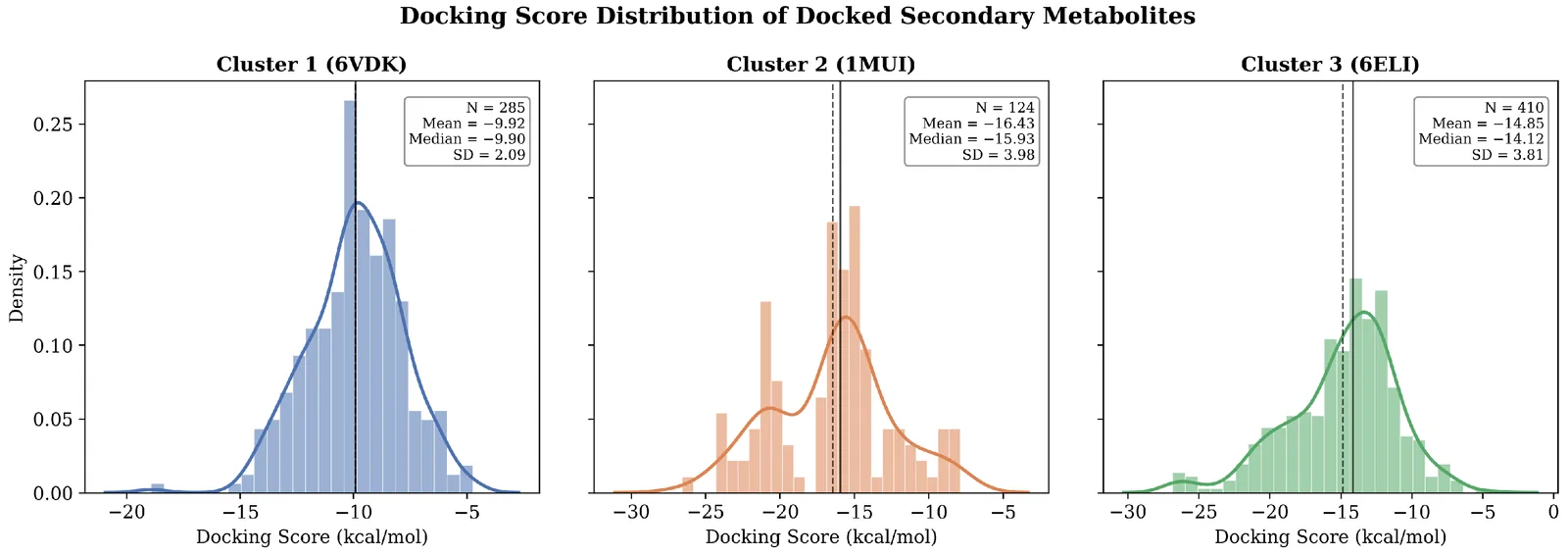
Docking score distributions of SMs docked against representative HIV-1 protein structures across the clusters.

The docking score distribution of the docked SMs is presented in Figure 5. For Cluster 1 (HIV-1 Integrase, 6VDK), a total of 285 SMs were docked against the corrected receptor and grid (Section 2.3), yielding docking scores ranging from −18.91 to −4.72 kcal/mol, with a mean of −9.92 ± 2.09 kcal/mol and a median of −9.90 kcal/mol. For Cluster 2 (HIV-1 Protease, 1MUI), 124 SMs were docked, producing docking scores ranging from −26.54 to −7.87 kcal/mol, with a mean of −16.43 ± 3.98 kcal/mol and a median of −15.93 kcal/mol. For Cluster 3 (HIV-1 Reverse Transcriptase, 6ELI), the largest set of 410 SMs was docked, with docking scores ranging from −26.83 to −4.59 kcal/mol. The mean docking score was −14.85 ± 3.81 kcal/mol, and the median was −14.12 kcal/mol. The three clusters were docked against structurally distinct targets with different pocket sizes and geometries, so these mean and range differences across clusters reflect target-specific scoring-function behavior rather than differential binding strength; docking-score magnitude is therefore compared only within a given target’s own cluster throughout this study, and is never used to infer that one target’s compounds bind more potently than another’s.

#### 2.5.2. Top-Performing Secondary Metabolites Identified by Docking

The top-performing SMs from each HIV-1 protein cluster were identified by ranking the compounds based on their SMINA docking scores. The five compounds with the lowest (most favorable) docking scores were selected as representative compounds (Table 5).

For Cluster C1 (integrase), docking against the extended intasome structure (PDB 6VDK), corrected to center the search grid on the validated dolutegravir-binding site (Section 2.3), identified several metabolites with predicted docking scores below −14 kcal/mol, with the top-ranked compound (CID C00002829) exhibiting a docking score of −18.91 kcal/mol.

Cluster C2 (protease) yielded the most negative docking scores of the three clusters (Section 2.5.1). Docking against PDB 1MUI identified multiple compounds with docking scores exceeding −24 kcal/mol, with the top-ranked metabolite (CID C00009310) exhibiting a docking score of −26.54 kcal/mol, consistent with the larger, deeper active-site pocket of HIV-1 protease rather than indicating stronger binding relative to the other two targets.

Similarly, Cluster C3 (reverse transcriptase) yielded several high-scoring candidates when docked against PDB 6ELI. All five top-ranked compounds displayed docking scores greater than −26 kcal/mol, indicating a consistently favorable binding profile for metabolites targeting the functional regions of reverse transcriptase captured by the representative structure.

These top-ranked docking results provide candidate SMs, prioritized within each target’s own cluster, for subsequent pharmacokinetic and physicochemical evaluations.

### 2.6. ADME and Bioavailability Analysis

#### 2.6.1. Cluster 1 ADME and Bioavailability Filtering

The strongest docking hits for Cluster 1 (integrase) against the corrected receptor/grid (Section 2.3) were compounds C00002829 (Hypericin), C00016684 (Complestatin; Chloropeptin II), C00006095 (6-*C*-Glucopyranosyldihydroquercetin), C00015144 (Complestatin B; Neuroprotectin B), and C00006268 (Spinosin). Among these compounds, C00002829 showed the lowest predicted docking score of −18.91 kcal/mol. Four of these five compounds were evaluable by SwissADME and showed poor drug-like characteristics, including bioavailability radar profiles extending beyond the optimal range, multiple violations of Lipinski’s rule of five, and low oral bioavailability scores (Figure S2, Supplementary Material S2). The fifth, C00015144, could not be evaluated at all: its SMILES representation (209 characters) exceeds SwissADME’s 200-character processing limit, so its exclusion reflects a platform limitation rather than a predicted pharmacokinetic liability. Notably, C00016684 (Complestatin) and C00015144 (Complestatin B) belong to the complestatin family of glycopeptides, which has been independently and experimentally confirmed as a class of HIV-1 integrase inhibitors, with reported activity against both strand-transfer catalysis and viral replication in infected cells [41]; that the docking protocol recovered these known integrase-active natural products among its strongest hits, despite their subsequent exclusion from the final candidate set (C00016684 on ADME/radar grounds, C00015144 as not evaluable by SwissADME), provides independent, literature-based support for the discriminative validity of the corrected docking pipeline beyond the docking scores alone.

Following the ranked ADME-screening procedure described in Section 4.7, three compounds were retained as the final candidates for Cluster 1: C00002145 (Camptothecin), C00052100 (11-Hydroxycamptothecin), and C00064112 (Hydroxycamptothecin).

**Figure 6:**
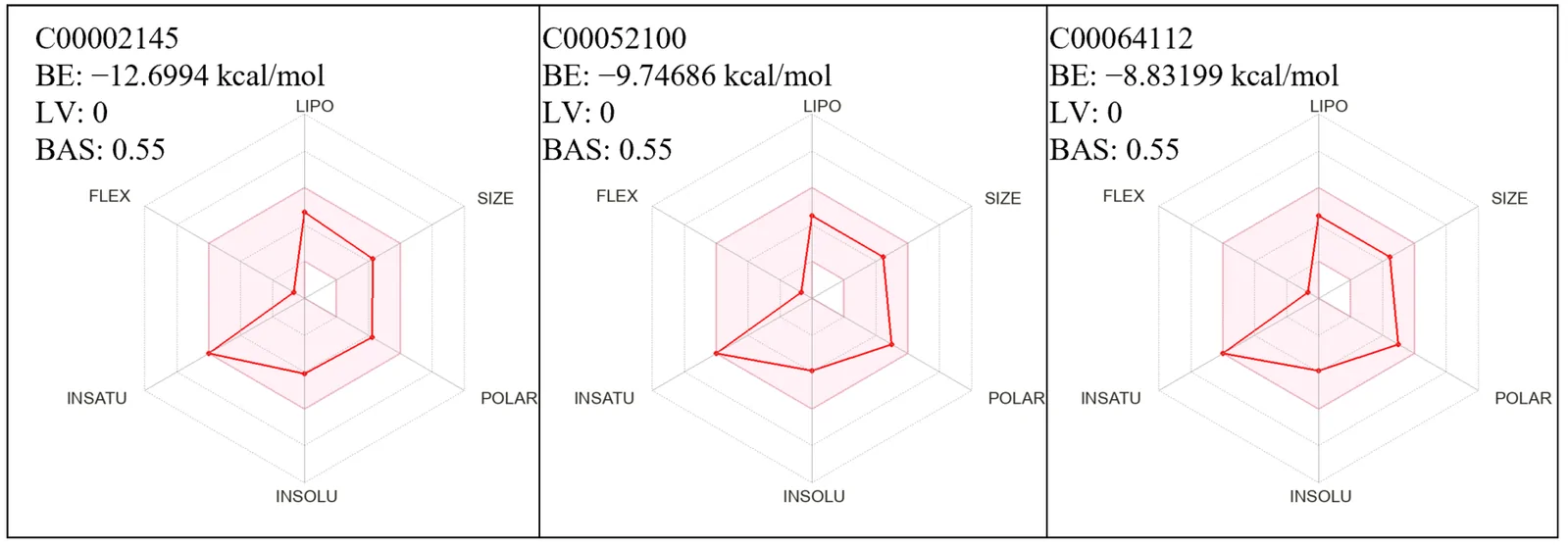
SwissADME bioavailability radar profiles of ADME-prioritized SMs for Cluster 1 (integrase).

These compounds exhibited favorable pharmacokinetic properties, including high gastrointestinal absorption, no Lipinski rule violations, and bioavailability scores of 0.55, indicating a better balance between predicted docking performance and drug-like properties. The SwissADME bioavailability radar profiles of these compounds are presented in Figure 6. Beyond the radar properties, extended SwissADME profiling (Table 6) indicated that C00002145 is predicted to inhibit three cytochrome P450 isoforms (CYP1A2, CYP2C9, CYP3A4) and a higher potential for drug–drug interactions than C00052100 and C00064112, which each inhibited only CYP1A2. ProTox 3.0 toxicity screening (Table 7) further indicated that all three compounds are predicted cytotoxic with high confidence (probability 0.96–0.98), consistent with camptothecin’s established mechanism as a topoisomerase I poison and the basis of its clinical use as a cytotoxic anticancer agent (as the derivatives irinotecan and topotecan); this is a substantial liability for repurposing as an antiviral, since cytotoxicity to host cells is generally undesirable outside oncology, and should be weighed heavily against this cluster’s otherwise favorable docking and bioavailability profile.

#### 2.6.2. Cluster 2 ADME and Bioavailability Filtering

For Cluster 2 (HIV-1 protease), the five highest-ranked metabolites based on docking scores could not be evaluated using SwissADME because their SMILES representations exceeded the 200-character processing limit.

Following the same ranked ADME-screening procedure (Section 4.7), three compounds were retained as the final candidates for Cluster 2: C00017264 (18-deoxycytochalasin H; L 696474), C00048932 (16-hydroxycarnosic acid), and C00036880 (carnosic acid).

**Figure 7:**
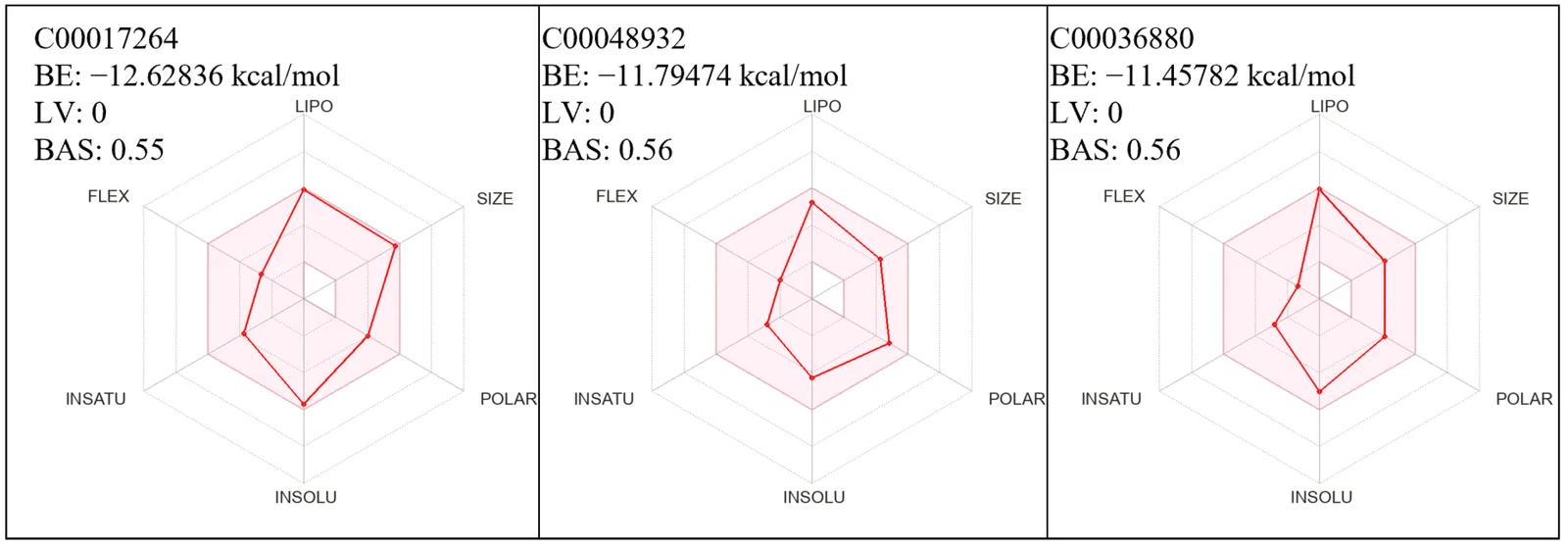
SwissADME bioavailability radar profiles of ADME-prioritized SMs for Cluster 2 (protease).

As shown in Figure 7, the three compounds showed bioavailability radar profiles within the optimal (pink) region, with no Lipinski violations and bioavailability scores ranging from 0.55 to 0.56. ProTox 3.0 screening (Table 7) flagged a single active endpoint per compound at moderate confidence: predicted hepatotoxicity for C00017264 (probability 0.60) and predicted hERG-related cardiotoxicity for both C00048932 and C00036880 (probabilities 0.61 and 0.56, respectively). C00017264 had the most favorable predicted acute oral toxicity of all nine candidates (LD50 4000 mg/kg, GHS Class 5).

#### 2.6.3. Cluster 3 ADME and Bioavailability Filtering

In the case of Cluster 3 (HIV-1 reverse transcriptase), the top-ranked SM, C00002268 (Tomatine), could not be evaluated using SwissADME because its SMILES representation exceeded the platform’s 200-character processing limit. Pharmacokinetic analysis of the remaining four leading compounds using SwissADME indicated that these compounds exhibited poor drug-like properties (Figure S3, Supplementary Material S2).

**Figure 8:**
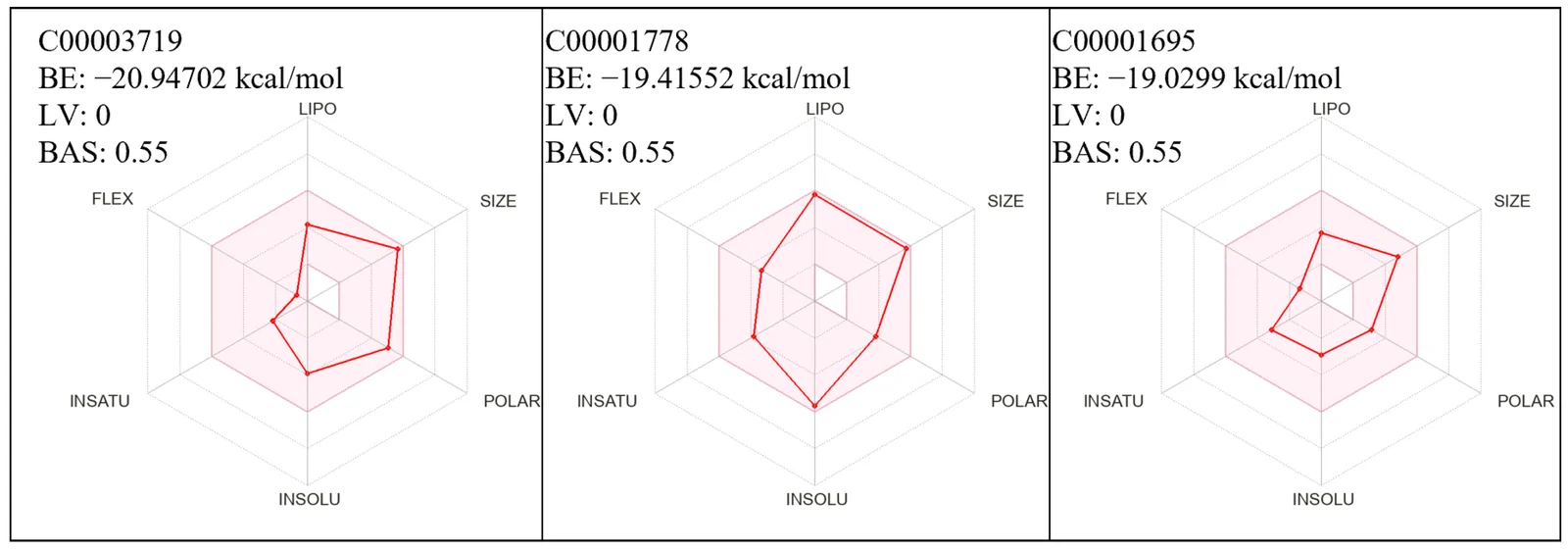
SwissADME bioavailability radar profiles of ADME-prioritized SMs for Cluster 3 (reverse transcriptase).

These results indicate that, as observed for the other clusters, docking-based prioritization alone was insufficient to identify pharmacokinetically optimal candidates for Cluster 3. Following the same ranked ADME-screening procedure (Section 4.7), three compounds were retained as the final candidates for Cluster 3: C00003719 (Limonin), C00001778 (Tubulosine), and C00001695 (Brucine). The physicochemical and pharmacokinetic profiles of these three compounds are shown in Figure 8.

As shown in Figure 8, these compounds maintained a balanced distribution of lipophilicity, size, polarity, solubility, flexibility, and saturation within an optimal drug-like space. Notably, despite being selected based on ADME criteria rather than docking rank alone, these metabolites also retained favorable predicted docking scores toward HIV-1 reverse transcriptase (−20.95 to −19.03 kcal/mol), indicating that pharmacokinetic optimization did not substantially compromise binding potential. Extended SwissADME profiling (Table 6) additionally indicated that C00001778 (Tubulosine) and C00001695 (Brucine) are predicted to be blood–brain barrier (BBB) permeant, unlike C00003719 or any of the six candidates from the other two clusters; both are alkaloids with documented central nervous system activity in the literature, which should be considered when prioritizing these compounds for further development, as it raises the possibility of off-target central nervous system exposure. ProTox 3.0 screening (Table 7) reinforces this concern for C00001695 specifically: it had the lowest predicted acute oral toxicity threshold among the reverse-transcriptase candidates, and the second-lowest of all nine candidates overall after the three integrase compounds (LD50 150 mg/kg, GHS Class 3, versus LD50 50 mg/kg for each integrase candidate), together with a predicted-active carcinogenicity flag, consistent with brucine’s known pharmacology as a toxic strychnos alkaloid. C00003719 showed a single predicted-active cardiotoxicity flag (probability 0.53). C00001778 was the only one of the nine candidates with no active flags on the ProTox endpoints screened here, despite its predicted BBB permeation; this reflects the absence of flags on this limited endpoint panel, not a demonstration of overall safety.

Table 6 summarizes this extended pharmacokinetic profiling—gastrointestinal absorption, BBB permeation, P-glycoprotein (P-gp) efflux, cytochrome P450 (CYP) isoform inhibition, four additional drug-likeness rule sets (Ghose, Veber, Egan, Muegge), and structural-alert screens (PAINS, Brenk)—for all nine final candidates across the three clusters.

All nine candidates showed high predicted gastrointestinal absorption and zero Lipinski violations, consistent with their selection via the bioavailability radar filter. Structural-alert screening flagged PAINS alerts for three compounds (C00048932, C00036880, C00001778) and Brenk alerts for five of the nine candidates; these are common, non-disqualifying flags for natural products with complex polycyclic scaffolds, but warrant medicinal-chemistry review before any experimental follow-up. Synthetic accessibility scores ranged from 3.81 (relatively easy) to 6.64 (C00017264, reflecting its large, densely functionalized cytochalasin scaffold).

The SwissADME-based analysis above evaluates pharmacokinetic and physicochemical drug-likeness but not toxicity. The nine candidates were finalized through the docking-plus-ADME-radar procedure alone (Section 4.7); toxicity was assessed afterward, using ProTox 3.0 [42] for predicted acute oral toxicity (median lethal dose, LD50) and five toxicological endpoints (hepatotoxicity, hERG-related cardiotoxicity, carcinogenicity, Ames-related mutagenicity, and cytotoxicity; Table 7), and is reported here as a disclosed caveat on this already-fixed candidate set, to inform rather than replace the experimental validation these predictions still require.

The clearest pattern is cluster-specific: all three integrase candidates (the camptothecin family) were predicted cytotoxic with high confidence, whereas the protease and reverse-transcriptase candidates each showed at most one active endpoint, at moderate confidence (0.53–0.61). C00001778 (Tubulosine) was the only candidate with no active toxicity flags on any of the five screened endpoints. Because these are computational structure–activity predictions rather than experimental measurements, they should be treated as hypothesis-generating flags for prioritizing subsequent in vitro toxicity assays, not as definitive safety assessments.

### 2.7. Comparative Analysis with FDA-Approved and Clinical HIV-1 Inhibitors

**Figure 9:**
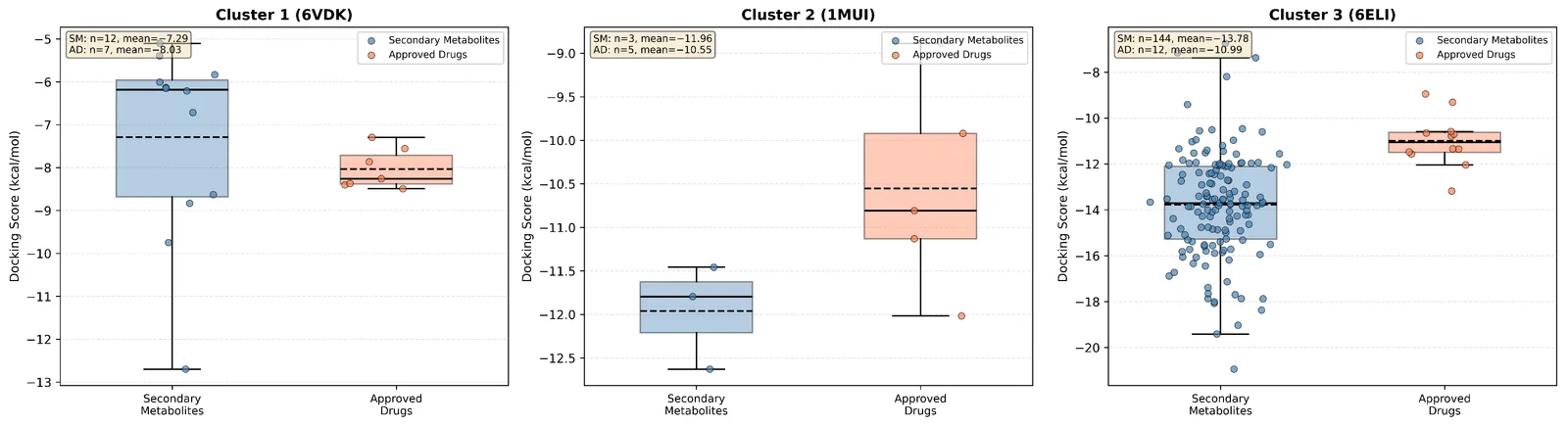
Box-and-strip plot comparison of docking scores between ADME-prioritized SMs (blue) and FDA-approved HIV-1 inhibitors (coral) across the three protein clusters. Each data point represents an individual compound in the dataset. Box plots display the median (solid line), mean (dashed line), interquartile range (box), and whiskers extending to 1.5× IQR. Docking was performed using SMINA against the representative structures 6VDK (cluster 1), 1MUI (cluster 2), and 6ELI (cluster 3). For cluster 3, the approved-drug set is restricted to the 12 NNRTIs mechanistically relevant to the 6ELI NNRTI-pocket structure (see main text for rationale). Lower docking scores indicate more favorable predicted binding.

To contextualize the docking performance of ADME-prioritized SMs, FDA-approved and clinical HIV-1 inhibitors retrieved from ChEMBL (Supplementary Material S2, Table S7) were docked against representative protein structures of each cluster using an identical protocol. The resulting docking score distributions are shown in Figure 9.

To statistically evaluate differences in docking scores between the ADME-prioritized SMs and FDA-approved drugs, a two-sided Mann–Whitney *U* test was employed as a nonparametric alternative because of the unequal sample sizes and the assumption of non-normality. Welch’s *t*-test was additionally performed to account for unequal variances between the groups. Effect sizes were quantified using Cohen’s *d*, where |d|<0.2 was considered negligible, 0.2≤|d|≤0.8 was considered small to medium, and |d|>0.8 was considered large. All statistical analyses were performed using SciPy v1.17.1 for Python 3.8 or higher. A significance threshold of p<0.05 was applied to all tests. Neither compared group is a random or independent sample in the classical statistical sense: the SM group is the deterministic output of the ranked docking-then-ADME selection procedure described above, not a random draw from the KNApSAcK library, and the approved/clinical-drug group is a fixed, mechanistically curated reference set. The significance tests below are therefore reported alongside, and secondary to, the descriptive statistics and effect sizes, which are the primary basis for interpreting these comparisons. The results of the statistical comparisons are presented in Table 8. The full descriptive statistics, including medians, ranges, and proportion analyses, are provided in Supplementary Materials S3, Table S15.

Among the three clusters, Cluster 3 (6ELI) exhibited the most striking differences between the two groups. The representative structure 6ELI is co-crystallized with rilpivirine within the non-nucleoside reverse-transcriptase inhibitor (NNRTI) allosteric pocket, and the docking grid for this cluster is centered on that same pocket. Nucleoside/nucleotide reverse-transcriptase inhibitors (NRTIs/NtRTIs) act at a different site (the polymerase active site, following intracellular phosphorylation) and are not expected to bind meaningfully at the NNRTI pocket; pooling them into the same statistical comparator as NNRTIs is therefore not mechanistically appropriate. Of the 31 FDA-approved/clinical reference compounds originally retrieved for this cluster, 12 are genuine NNRTIs and the remaining 19 are NRTIs/NtRTIs; the comparison below uses the 12 NNRTIs as the primary comparator, with the NRTI subset reported separately for context rather than as a valid comparator.

The ADME-prioritized SMs (n=144) achieved a mean docking score of −13.78±2.31 kcal/mol, which was substantially lower than that of the NNRTI reference set (n=12, mean =−10.99±1.09 kcal/mol). This difference was highly significant according to both the Mann–Whitney *U* test (U=191.0, $p=7.73\times 10^{-6}$) and Welch’s *t*-test (t=−7.319, $p=4.71\times 10^{-7}$), with a large effect size (Cohen’s d=−1.540); this is also the most imbalanced of the three comparisons (n=144 versus n=12), and the large SM sample size increases statistical power to detect even a modest difference, so the large effect size, not the small *p*-value alone, is the more informative indicator of the magnitude of this difference. A total of 62.5% of the SMs (90 out of 144) achieved docking scores lower than that of the best-performing NNRTI (−13.18 kcal/mol), and 92.4% (133 out of 144) scored below the mean docking score of the NNRTI set. For context, the excluded NRTI/NtRTI subset (n=19) scored considerably worse overall (mean =−8.58±1.11 kcal/mol) than both the SMs and the NNRTIs, consistent with these compounds not being expected to bind the NNRTI pocket; this comparison is reported for completeness only and should not be interpreted as evidence of relative potency, since NRTIs require intracellular triphosphorylation and act through an entirely different binding mode not captured by this docking protocol.

For Cluster 2 (1MUI), the SMs (n=3) exhibited a mean docking score of −11.96±0.49 kcal/mol, compared with −10.55±1.07 kcal/mol for the approved drugs (n=5). Although the effect size was large (Cohen’s d=−1.692) and all three SMs scored below the mean of the approved drugs, the difference did not reach statistical significance (Mann–Whitney *U* test: p=0.143; Welch’s *t*-test: p=0.070), likely because of the limited sample size.

In Cluster 1 (6VDK), redocked against the corrected receptor/grid (Section 2.3), no significant difference was observed between the SMs (n=12, mean =−7.29±2.16 kcal/mol) and the approved drugs (n=7, mean =−8.03±0.43 kcal/mol), as confirmed by both the Mann–Whitney *U* test (p=0.261) and Welch’s *t*-test (p=0.289), with a small-to-medium effect size (Cohen’s d=0.480) favoring the approved drugs. Nonetheless, the SMs exhibited considerably greater variability in docking scores, with 33% (4 out of 12) achieving scores better than that of the best-performing approved drug (−8.49 kcal/mol, bictegravir).

Overall, these results indicate that ADME-prioritized SMs from KNApSAcK exhibit competitive or, for the 6ELI target, more favorable predicted docking scores than established HIV-1 inhibitors under this docking protocol. Their consistently favorable docking performance, combined with acceptable pharmacokinetic profiles confirmed through ADME filtering, identifies these natural product-derived compounds as computationally prioritized candidates warranting experimental validation against HIV-1 protein targets, rather than as confirmed lead compounds at this stage.

### 2.8. Compound-Level Comparison Against Co-Crystallized Reference Inhibitors

Group-level statistical comparisons summarize distributions of docking scores but do not, by themselves, demonstrate that any individual SM binds more favorably than a specific, biologically relevant reference compound at the same site. As a complementary, compound-level check, the top-ranked ADME-prioritized SM for each cluster was compared directly against that structure’s own co-crystallized ligand, redocked into its native site under an identical protocol (Section 2.3), the most direct, mechanistically matched reference available for each target (Table 9).

In all three cases, the top-ranked SM scored more favorably than its target’s own co-crystallized reference ligand under the identical docking protocol. This comparison should be read alongside the docking-validation results (Section 2.3): none of the three redocked reference ligands reproduced their crystallographic pose within the conventional 2 Å threshold, so these ligand scores (like all docking scores reported in this study) carry the same pose-accuracy uncertainty already disclosed, and are not a substitute for the group-level statistical comparisons above. The value of this compound-level check is narrower and more specific: it confirms that each top-ranked SM obtains a more favorable docking score than a single, mechanistically appropriate, directly relevant reference compound under the exact same conditions used to score it, rather than only a heterogeneous reference-drug group.

## 3. Discussion

### 3.1. Overview of Binding Interaction Profiles

**Figure 10:**
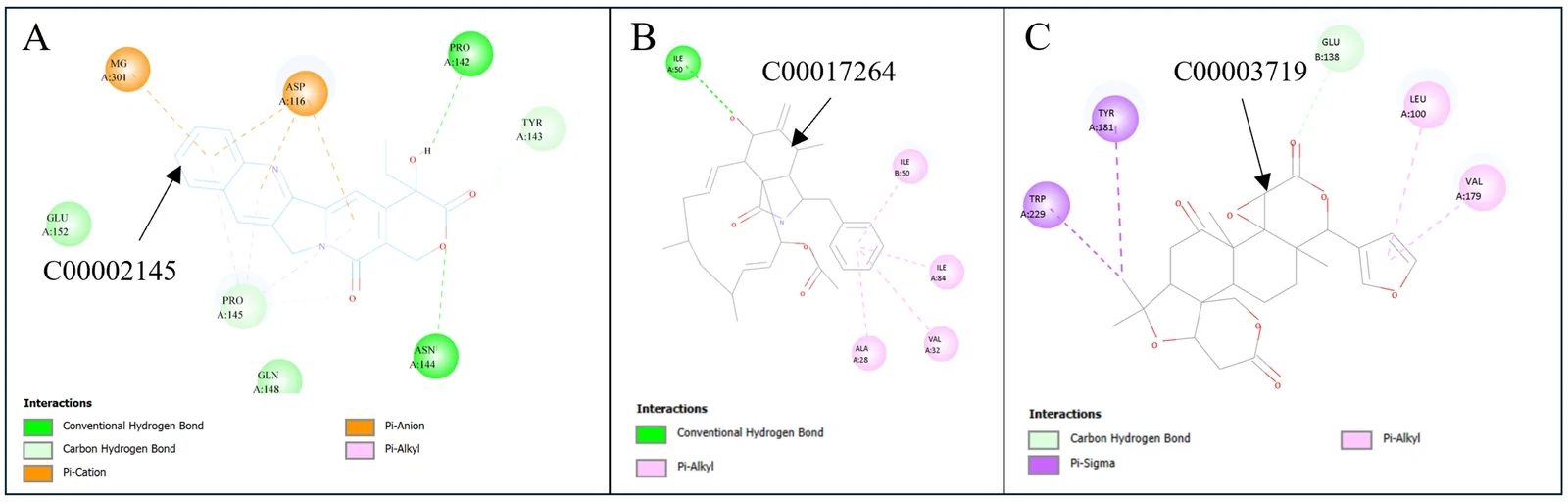
Two-dimensional interaction diagrams of the top-ranked metabolites binding to the three HIV-1 protein targets, generated from the docking pose and presented as predicted contacts rather than validated binding modes (Section 2.3). (**A**) C00002145 binding to HIV-1 integrase (PDB: 6VDK), showing predicted conventional hydrogen bonds with ASN144 and PRO142, carbon–hydrogen bonds with TYR143 and PRO145, a π–cation interaction with the catalytic ${\mathrm{Mg}}^{2+}$ ion MG301, π–anion interactions with the DDE-triad residue ASP116, and hydrophobic π–alkyl contacts with PRO145. (**B**) C00017264 binding to HIV-1 protease (PDB: 1MUI), displaying a predicted conventional hydrogen bond with ILE50 and hydrophobic interactions with ALA28, VAL32, ILE84, and ILE50. (**C**) C00003719 binding to HIV-1 reverse transcriptase (PDB: 6ELI), showing a predicted carbon–hydrogen bond with GLU138, π–σ interactions with TYR181 and TRP229, and hydrophobic contacts with LEU100 and VAL179. Residues are represented as labeled colored circles, and interaction types are color-coded according to the legends shown in each panel. Dashed lines indicate specific interactions, with distances reported in Å.

Among the prioritized final candidates, molecular docking analysis identified distinct binding profiles across the three HIV-1 protein targets: Cluster 1 (HIV-1 integrase, PDB: 6VDK), redocked against the corrected receptor/grid (Section 2.3), yielded docking scores ranging from −12.70 to −8.83 kcal/mol; Cluster 2 (HIV-1 protease, PDB: 1MUI) produced scores between −12.63 and −11.46 kcal/mol; and Cluster 3 (HIV-1 reverse transcriptase, PDB: 6ELI) yielded docking scores ranging from −20.95 to −19.03 kcal/mol (Supplementary Materials S3, Tables S12–S14); as with the full-library score distributions (Section 2.5.1), these more negative values reflect the deep reverse-transcriptase NNRTI pocket rather than a stronger binding signal relative to the other two targets. These docking scores are, for the reverse-transcriptase target, more favorable than those of the mechanistically matched reference inhibitors under the same protocol; as computational scoring-function outputs, they support candidate prioritization but do not by themselves establish comparable binding affinity. The 2D interaction diagrams generated from BIOVIA Discovery Studio Visualizer v25.1.0.24284 (Figure 10A–C) reveal that the prioritized candidates engage the targets through hydrogen bonds, hydrophobic interactions, and aromatic contacts, which are consistent with the interactions of known inhibitors. The residue-level descriptions in this section are derived from single, unrefined docking poses; given the pose-reproduction limitations established in Section 2.3, they are presented as hypothetical docking poses rather than validated binding modes, and should be read as illustrating contact patterns consistent with the predicted docking score, not as confirmed structural detail.

### 3.2. Cluster 1 (HIV-1 Integrase): Top Candidate C00002145

**Figure 11:**
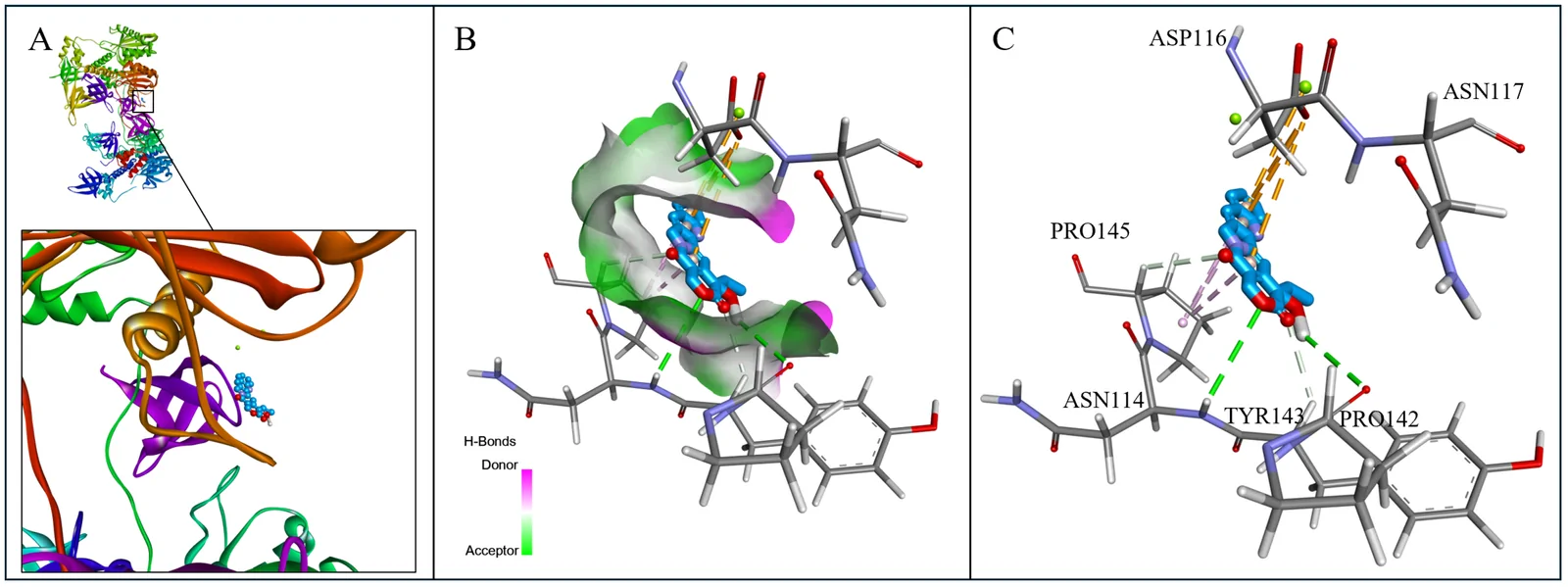
3D structural visualizations of the top-ranked secondary metabolite C00002145 docked into the corrected HIV-1 integrase active site (PDB ID: 6VDK; Section 2.3). (**A**) Ribbon representation of the overall protein–ligand complex, with an inset showing the location of the binding pocket. (**B**) Surface representation of the active site depicting the shape complementarity of the ligand within the pocket and highlighting the hydrogen-bond donor and acceptor surfaces. (**C**) Detailed 3D noncovalent interaction network illustrating the predicted binding mode. Hydrogen bonds (green dashed lines) connect the ligand to ASN144, PRO142, TYR143, and PRO145. Electrostatic contacts (orange/red dashed lines) indicate predicted contact with the catalytic ${\mathrm{Mg}}^{2+}$ ion MG301 through a π–cation interaction and the DDE-triad residue ASP116 through π–anion interactions, while hydrophobic π–alkyl contacts with PRO145 are predicted to further stabilize the complex.

C00002145 (Camptothecin) achieved the highest integrase docking score (−12.70 kcal/mol) among the cluster 1 candidates, with a predicted binding mode combining conventional and carbon–hydrogen bonding, electrostatic contacts with the catalytic metal-coordination sphere, and hydrophobic stabilization (Figure 10A and Figure 11; Supplementary Materials S2, Table S9). The docked pose places conventional hydrogen bonds with ASN144 (2.95 Å) and PRO142 (2.76 Å), and carbon–hydrogen bonds with TYR143 (2.33 Å) and PRO145 (2.95 Å), near the entrance of the active site. Electrostatically, the pose suggests contact between the ligand and the catalytic ${\mathrm{Mg}}^{2+}$ ion MG301 through a π–cation interaction (4.01 Å) and the DDE-triad residue ASP116 through three π–anion contacts (3.52–4.26 Å), the same catalytic aspartate that, together with ASP64 and GLU152, coordinates the two-${\mathrm{Mg}}^{2+}$ centers essential for strand-transfer catalysis (Introduction). Three additional π–alkyl hydrophobic contacts with PRO145 (3.88–4.10 Å) complete the predicted binding mode, consistent with the metal-coordination-proximal mechanism exploited by clinically approved INSTIs rather than the peripheral pocket-lining contacts predicted prior to correction of the docking grid.

This predicted interaction profile resembles known integrase strand-transfer inhibitors (INSTIs), which characteristically chelate the two active-site ${\mathrm{Mg}}^{2+}$ ions and engage the DDE catalytic triad alongside hydrogen bonds and hydrophobic contacts with surrounding pocket residues [8,9,43,44]; such catalytic-site engagement, together with aromatic/hydrophobic stacking, has similarly been linked computationally to INSTI binding [45]. The second- and third-ranked candidates, C00052100 and C00064112, show predicted contacts directed at the same catalytic machinery through different residue subsets (Supplementary Materials S2, Table S9): C00052100’s pose adds a contact with the DDE-triad residue GLU152 to hydrogen bonds shared with C00002145, and C00064112’s pose is notable for a short contact (2.28 Å) to the second catalytic ${\mathrm{Mg}}^{2+}$ ion, MG302, alongside contacts with ASP64 and ASP116. Taken together, all three prioritized integrase candidates are predicted to converge on the same catalytic ${\mathrm{Mg}}^{2+}$/DDE-triad region targeted by clinically used INSTIs, despite differing peripheral contacts, offering a mechanistically coherent rationale for their prioritization beyond docking score alone. Notably, all three candidates, the camptothecin family, were also independently mapped to the reverse-transcriptase cluster via BindingDB. This is consistent with an independently documented, topoisomerase-I-mediated route by which camptothecin affects HIV-1 reverse transcription, distinct from any direct reverse-transcriptase binding predicted by docking here: cellular topoisomerase I enhances HIV-1 reverse-transcriptase activity, an effect specifically blocked by camptothecin [46], and camptothecin itself blocks HIV-1 infection in cell culture, as measured by reduced reverse-transcriptase activity [47]. This is a literature-consistent observation about the compound-first pipeline’s behavior, not evidence that the docking predictions themselves capture this mechanism.

### 3.3. Cluster 2 (HIV-1 Protease): Top Candidate C00017264

**Figure 12:**
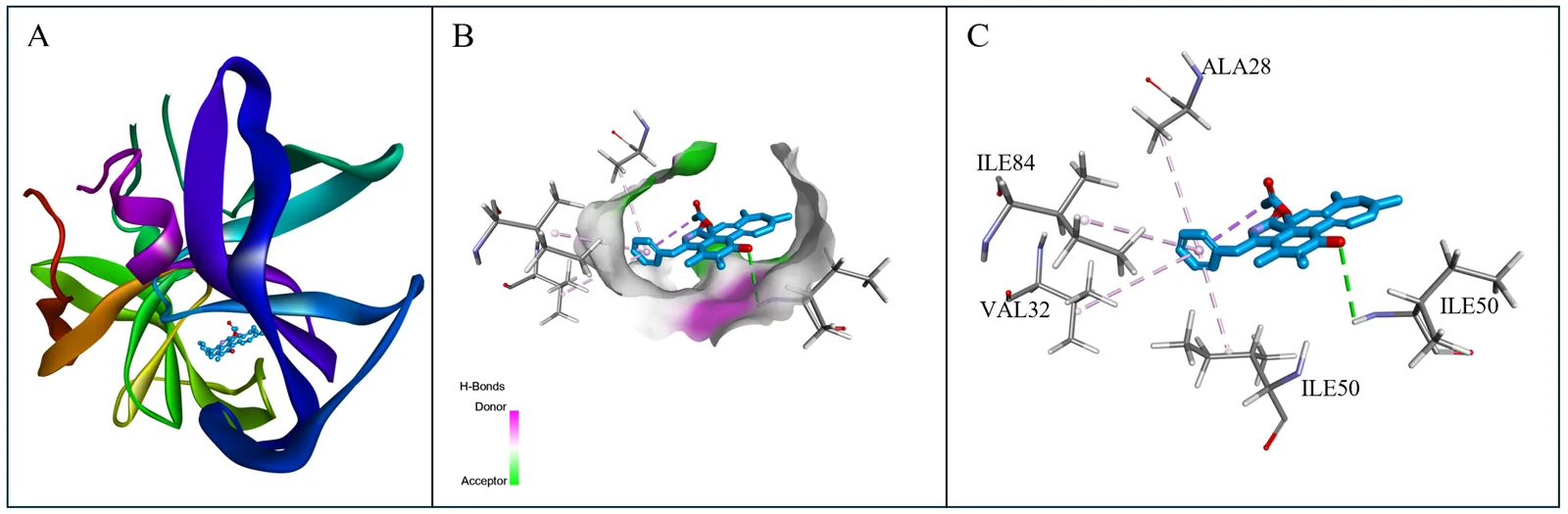
3D structural visualizations of the top-ranked secondary metabolite C00017264 docked into the HIV-1 protease active site (PDB ID: 1MUI). (**A**) Ribbon representation of the overall protein–ligand complex. (**B**) Surface representation of the active site, illustrating the high degree of shape complementarity and a predicted hydrophobic cage enveloping the ligand. (**C**) Detailed 3D noncovalent interaction network. A single conventional hydrogen bond (green dashed line) connects the ligand to the protease flap residue ILE50. The predominantly hydrophobic predicted binding mode is stabilized by π–alkyl interactions (purple/pink dashed lines) with the S-pocket residues ALA28, VAL32, ILE84, and ILE50.

C00017264 (18-Deoxycytochalasin H; L 696474) achieved the most favorable docking score in the protease cluster (−12.63 kcal/mol), with a predicted binding mode that is predominantly hydrophobic (Figure 10B and Figure 12; Supplementary Materials S2, Table S10). The docked pose places one conventional hydrogen bond with ILE50 (3.02 Å) (Figure 12C) and four π–alkyl hydrophobic contacts with ALA28 (4.81 Å), VAL32 (5.32 Å), ILE84 (4.75 Å), and ILE50 (4.06 Å). As visualized in the surface representation (Figure 12B), these predicted contacts suggest a tight hydrophobic cage around the ligand.

This hydrophobic-dominant profile is consistent with structural analyses showing that HIV-1 protease inhibitors derive substantial binding energy from hydrophobic packing within the S1/S2 subsites [48,49], and docking studies of Food and Drug Administration (FDA)-approved protease inhibitors have similarly linked hydrophobic contacts with flap residues, including ILE50, and S-pocket residues to binding affinity [50]. ILE50 sits at the tip of the flexible flap (residues approximately 43–58) that opens and closes over the substrate-binding cleft in the catalytic mechanism outlined in the Introduction; contacts at this position would be mechanistically significant if the predicted pose is correct, since flap closure over a bound ligand is a prerequisite for occluding the active site and blocking substrate access, independent of any direct interaction with the Asp25/Asp25′ catalytic dyad itself [10]. The relatively long hydrogen-bond distance (3.02 Å) suggests that hydrophobic interactions dominate the docking score of this metabolite. The second- and third-ranked candidates, C00048932 and C00036880, show similar predicted hydrophobic engagement, with hydrogen-bond-rich and mixed contact patterns (Supplementary Materials S2, Table S10).

### 3.4. Cluster 3 (HIV-1 Reverse Transcriptase): Top Candidate C00003719

**Figure 13:**
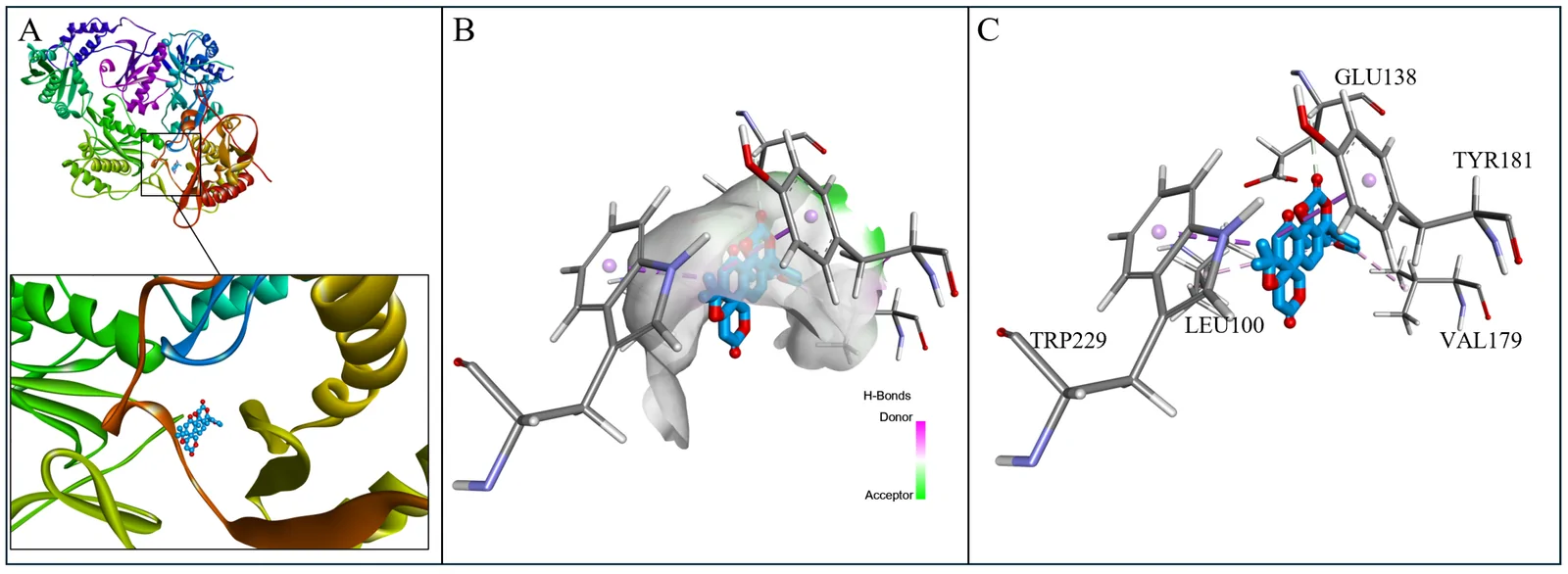
3D structural visualizations of the top-ranked secondary metabolite C00003719 docked into the HIV-1 reverse-transcriptase binding site (PDB ID: 6ELI). (**A**) Ribbon representation of the overall protein–ligand complex, featuring an inset showing the location of the ligand within the binding pocket. (**B**) Surface representation of the active site, illustrating the shape complementarity of the ligand within the deep hydrophobic pocket characteristic of non-nucleoside reverse-transcriptase inhibitors (NNRTIs). (**C**) Detailed 3D noncovalent interaction network. A predicted carbon–hydrogen bond connects the ligand to GLU138, whereas the predominantly aromatic and hydrophobic predicted binding mode is stabilized by π–σ interactions with the key aromatic residues TYR181 and TRP229 and by π–alkyl contacts with LEU100 and VAL179.

C00003719 achieved the most negative docking score of the nine final candidates (−20.95 kcal/mol, Section 2.5.1), a magnitude driven in part by the deep reverse-transcriptase NNRTI pocket rather than necessarily indicating the strongest binding relative to the integrase and protease candidates, with a predicted binding mode combining weak hydrogen bonding and strong aromatic interactions (Figure 10C and Figure 13; Supplementary Materials S2, Table S11). The docked pose places one carbon–hydrogen bond with GLU138 (2.50 Å) (Figure 13C), two π–σ interactions with TYR181 (3.80 Å) and TRP229 (3.76 Å), and two π–alkyl hydrophobic contacts with LEU100 (5.30 Å) and VAL179 (4.26 Å) (Supplementary Materials S2, Table S11).

Because the retrospective DUD-E enrichment analysis found essentially no discriminative validity for this target (ROC-AUC = 0.529, Section 2.4), consistent with 6ELI’s comparatively poor pose-reproduction accuracy (Section 2.3), the reverse-transcriptase candidate prioritization is substantially less reliable than that obtained for integrase and protease, and the interaction-level interpretations that follow should be read as particularly uncertain. The predicted interaction pattern resembles that of non-nucleoside reverse-transcriptase inhibitors (NNRTIs), which bind a hydrophobic pocket formed by aromatic residues, including TYR181, TYR188, and TRP229 [7,15,51]; computational studies have linked π–σ and π–π interactions with these aromatic residues to NNRTI potency [7]. TYR181 and TRP229 are not themselves catalytic residues; as outlined in the Introduction, this allosteric pocket sits roughly 10 Å from the polymerase active site (catalytic triad ASP110/ASP185/ASP186), and known NNRTIs act by stabilizing a pocket conformation that mechanically distorts the adjacent catalytic-triad geometry rather than by directly competing with the nucleic acid substrate [7]; the predicted contacts at this position are consistent with, but do not themselves confirm, this mechanism. The second-ranked candidate, C00001778, and the third-ranked RT candidate, C00001695, show similarly favorable docking scores and are predicted to engage the same aromatic residues through π-driven contacts (Supplementary Materials S2, Table S11), consistent with this interaction network’s relevance across the cluster’s top candidates.

### 3.5. Comparison with Known Inhibitors

The predicted binding profiles of the prioritized metabolites resemble those of established HIV-1 inhibitors. For integrase, the combination of hydrogen bonding and aromatic stacking predicted for C00002145 resembles the binding mode of raltegravir and related integrase strand-transfer inhibitors (INSTIs), which use aromatic interactions with active-site residues and viral DNA for stabilization [8,9,45,52]. The absence of metal-chelating groups, which are typical of INSTIs, from this predicted pose suggests that C00002145 may employ a different inhibitory mechanism or require structural modification to achieve optimal activity [9].

The docked pose for C00017264 suggests a hydrophobic-dominant binding mode, similar to inhibitors that exploit hydrophobic contacts within the S pockets, whereas C00048932’s predicted pose shows extensive hydrogen bonding with the backbone atoms of ASP29 and ASP30, resembling the design strategy of darunavir and bis-THF inhibitors [10,53]. Docking studies of FDA-approved protease inhibitors have reported similar interaction patterns involving these residues [11].

For reverse transcriptase, the docked poses of the three top-ranked candidates collectively place them in contact with the same aromatic-residue network characteristic of NNRTI binding [7] (TYR181, TYR188, and TRP229), though the specific residues contacted varied by compound: C00003719’s pose contacts TYR181 and TRP229, while C00001778 and C00001695 additionally or alternatively contact TYR188. Their docking scores, ranging from −20.95 to −19.03 kcal/mol, were more negative than those of the integrase and protease candidates, a difference attributable to the deep and well-defined hydrophobic pocket of the NNRTI-binding site (Section 2.5.1) rather than to genuinely stronger inhibitory potential; these reverse-transcriptase interaction predictions warrant particular caution, given the weak discriminative validity found for this target in Section 2.4.

### 3.6. Limitations and Future Directions

However, this docking analysis has several limitations. First, the docking scores are empirical outputs of a single scoring function and may not accurately rank compounds according to their experimental binding affinities [17,50,54]. Second, rigid-receptor docking does not account for protein flexibility, which can substantially affect binding modes and predicted docking scores [55]. Third, the analysis did not consider metal coordination, which is critical for integrase inhibition, or water-mediated interactions, both of which can influence inhibitor potency [56]. Fourth, docking alone cannot predict the inhibition mechanism or account for resistance-associated mutations that alter the binding pockets.

Redocking validation (Section 2.3) further indicated that none of the three representative structures reproduced their crystallographic ligand pose within the conventional ≤2 Å threshold, and that the fpocket-derived grid originally selected for the integrase screening was displaced by nearly 29 Å from the true dolutegravir-binding site, most likely because the protein-only receptor preparation omits the viral DNA strands that form part of the intasome active site. This grid was subsequently corrected (re-centered on the native ligand, with the Mg^2+^ ions restored) and the entire cluster-1 library redocked before any integrase results elsewhere in this study were generated, so the 29 Å displacement itself does not carry through to the reported results; however, the correction fixed only the grid’s spatial placement and the ion content, not the underlying structural omission: the viral DNA strands remain absent from the receptor used for every reported integrase result. The integrase (6VDK) docking results in this study therefore carry somewhat more uncertainty than the protease and reverse-transcriptase results for two compounding reasons: the corrected grid was set directly from the native ligand’s coordinates rather than independently validated by successful pose reproduction, and the receptor itself remains a structurally incomplete, DNA-free approximation of the true intasome active site. Results should be interpreted only as a coarse, redundancy-reducing prioritization signal rather than as evidence of a specific, structurally validated binding mode. SMINA/Vina’s scoring function also has no explicit term for metal–ligand chelation, so restoring the Mg^2+^ ions to the receptor corrects the grid-placement/steric context but does not add genuine metal-coordination scoring. Because all KNApSAcK candidates and reference compounds were docked under an identical, unvalidated-for-absolute-accuracy protocol within each target, relative rankings remain internally consistent for the purpose of compound prioritization, but the absolute docking poses and docking scores reported here should not be taken as structurally confirmed. This internal-consistency argument establishes only that compounds within a target were compared on equal footing; it does not by itself establish that the resulting ranking has real discriminative meaning rather than being merely self-consistent. The retrospective DUD-E enrichment analysis (Section 2.4) provides the actual evidence on this point, and shows that this distinction matters in practice: discriminative validity is well supported for integrase and protease (ROC-AUC 0.845 and 0.783) but not for reverse transcriptase (ROC-AUC 0.529), so the internal-consistency argument alone should not be relied upon, particularly for the reverse-transcriptase cluster.

A separate concern is that the representative structures were not selected using a cluster-derived criterion: 6VDK and 1MUI were chosen based on prior literature precedent, and 6ELI on crystallographic resolution, rather than on their centrality within the sequence-similarity cluster. To evaluate whether this affected the prioritized candidates, we computed each structure’s cluster medoid – the member with the highest mean pairwise sequence identity to all other structures in its own cluster, from the pre-threshold alignment network underlying the DPClusSBO clustering. This analysis showed that 6VDK and 1MUI are themselves atypical members of their clusters (ranked 134/135 and 104/117 by mean intra-cluster identity, respectively), whereas 6ELI is already close to its cluster medoid (ranked 5/42). As a robustness check, the three ADME-prioritized final candidates for integrase and protease were redocked against each cluster’s top medoid structure (4CJR for integrase; 7LE4 for protease, co-crystallized with darunavir) using an identical protocol (Table 10).

For integrase, the ranking of the three candidates was identical at both structures (C00002145 > C00052100 > C00064112), and absolute scores remained within 1.2 kcal/mol of the original values, indicating that the prioritization is robust to the choice between 6VDK and its cluster medoid despite 6VDK’s atypical position within the cluster. For protease, however, the ranking was not preserved: C00017264, the top-ranked candidate at 1MUI, dropped to third at the medoid structure 7LE4 (score shift of +3.77 kcal/mol, the largest of the six comparisons), while C00048932 became the top-ranked candidate. This is consistent with the substantial conformational variability documented for HIV-1 protease flap dynamics and ligand-bound active-site geometry [35], and indicates that the protease prioritization is more sensitive to the specific representative structure chosen than the integrase or reverse-transcriptase results. This sensitivity is reported as an additional, structure-dependent source of uncertainty for the protease candidates, distinct from the docking-validation limitations discussed above.

Representative structures were selected for biological and crystallographic reliability (literature precedent for 6VDK and 1MUI, crystallographic resolution for 6ELI) rather than for centrality within the sequence-similarity network, even though medoid centrality was directly computable from the same alignment data underlying the clustering. Global sequence identity, the basis of both the DPClusSBO clustering and of medoid centrality, does not guarantee similarity in binding-pocket geometry, conformational state, or resistance-associated mutations between cluster members. A structure’s network centrality is therefore not a reliable proxy for how well it represents the cluster’s ligand-binding behavior, and prioritizing receptor reliability over network centrality reflects this limitation rather than an oversight. Reducing 295 individual HIV-1 protein chains to three representative structures trades comprehensive structural-diversity coverage for computational tractability throughout this framework, not only at the clustering step. The medoid-sensitivity results above, protease sensitive but integrase and reverse transcriptase comparatively robust, demonstrate that this trade-off carries real, target-dependent consequences rather than a purely theoretical one.

Full ensemble docking, defined here as docking the entire compound library against multiple structures per cluster rather than against a single representative structure, was not pursued. Clustering exists specifically to replace redundant multi-structure docking with one representative structure per cluster; docking the full compound library (285, 124, and 410 SMs, respectively) against every cluster member, up to 135 structures for integrase alone, would reintroduce at a much larger scale the redundancy this framework is designed to eliminate. The medoid-sensitivity analysis above provides a proportionate robustness check at the appropriate scale: it redocks only the final, ADME-prioritized candidates rather than the full library, and already demonstrates the pattern a full ensemble study would be expected to show, structure-dependent sensitivity for protease and robustness for integrase. Systematic ensemble docking across all cluster members remains a valid direction for future work at a different scale and with a different objective, characterizing intra-cluster structural variability in its own right, rather than a gap in how the present study uses clustering to reduce, rather than reintroduce, redundant screening.

A further limitation concerns the depth of binding-mode validation for the final prioritized candidates. The method-level validation performed in this study, redocking against native ligands, Ramachandran structural-quality checks, the medoid-sensitivity analysis above, and the retrospective DUD-E enrichment analysis (Section 2.4), establishes that the docking protocol and target-selection procedure carry genuine discriminative validity for integrase and protease and identifies where that validity does not hold (reverse transcriptase), but none of this extends to molecular dynamics (MD) simulation or MM-GBSA/MM-PBSA binding free-energy refinement for the individual prioritized candidates. Such analyses are the appropriate next step for testing whether a specific candidate’s binding mode remains stable beyond a single static docking pose, but they validate individual compounds rather than the prioritization framework itself, which is the primary contribution of this study; MD/MM-GBSA on the three top candidates would only speak to those particular compounds, not to whether the underlying cluster-guided, redundancy-aware screening strategy generalizes. Their omission here therefore reflects a deliberate scope boundary rather than an oversight, and their inclusion would not alter the hypothesis-generating status already declared for every computational output in this study: MD and MM-GBSA remain in silico approximations that, like docking itself, ultimately require biochemical or structural confirmation. We identify MD/MM-GBSA-based refinement of the nine prioritized candidates as a concrete direction for follow-up work building on this framework.

Despite these limitations, the computational results identified natural product scaffolds with interaction profiles resembling those of known HIV-1 inhibitors. C00002145 for integrase, C00017264 for protease, and C00003719 for reverse transcriptase emerged as the top computationally prioritized candidates based on their favorable docking scores and interaction patterns consistent with those of established inhibitors. The diversity of the observed binding modes, ranging from hydrogen-bond-rich interactions for C00048932 to hydrophobic-dominant interactions for C00017264 and aromatic-rich interactions for C00003719, suggests that multiple chemical scaffolds may merit consideration as starting points for further inhibitor development, pending experimental confirmation. Experimental validation through biochemical assays and structural studies is required to confirm the predicted binding modes, determine the actual ligand-binding affinities, and assess inhibitory activity. More broadly, that this compound-first, BindingDB-mediated screening strategy yielded internally consistent, mechanistically interpretable candidates supports its viability as a complement to the protein-first direction validated previously for SARS-CoV-2 [13], underscoring the transferability of sequence-similarity-guided target organization across screening directions and viral systems.

## 4. Materials and Methods

**Figure 14:**
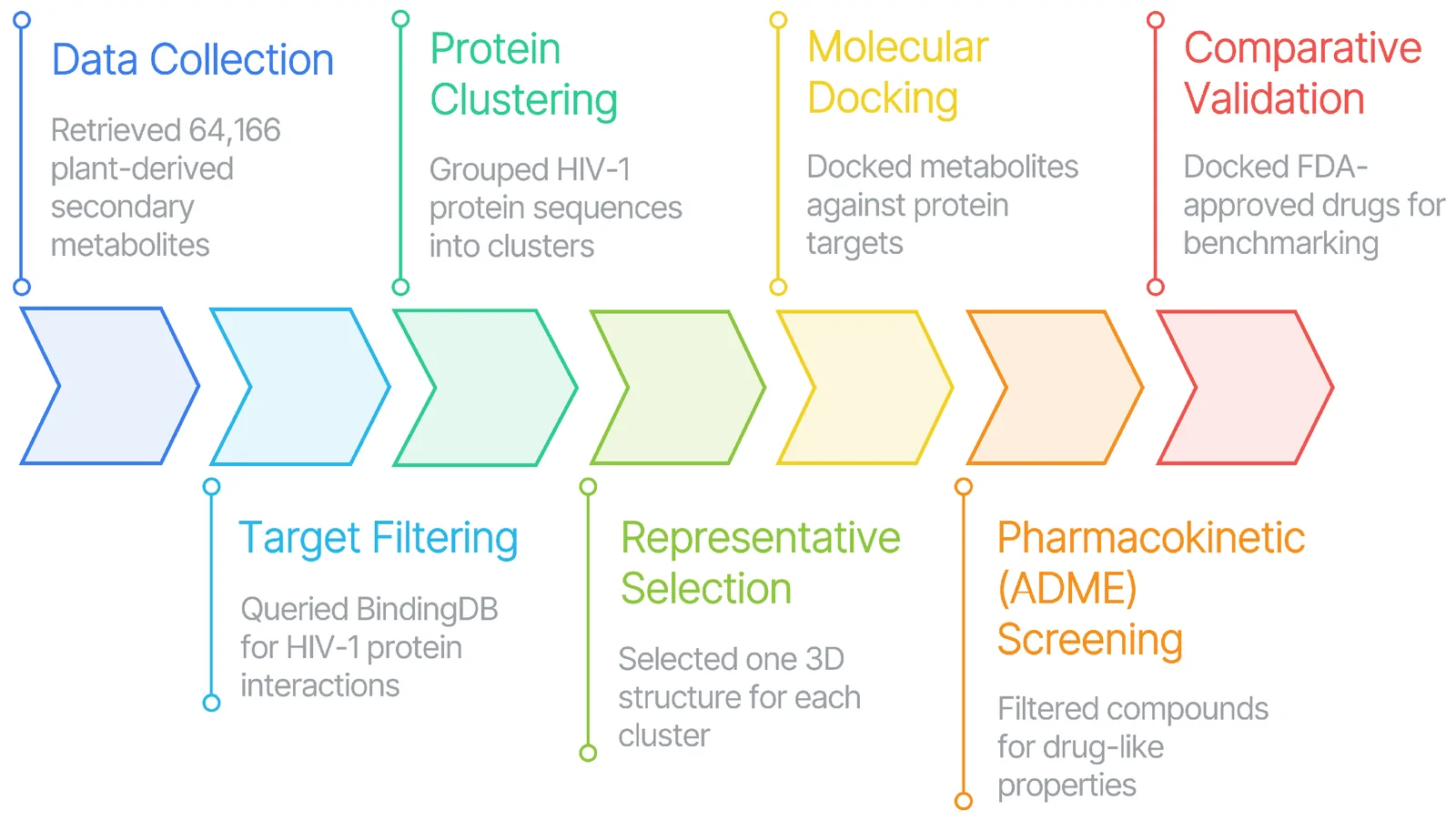
Overall computational workflow for cluster-guided prioritization of natural product candidates against HIV-1.

The systematic workflow employed in this study is illustrated in Figure 14, which outlines the major computational stages from SM collection to comparative analysis. Each methodological component is described in detail below.

### 4.1. Secondary Metabolite Dataset Acquisition and SMILES Conversion

A comprehensive dataset of 64,166 SMs was obtained in MOL file format from the KNApSAcK Family Database (https://www.knapsackfamily.com/KNApSAcK/, retrieved 22 September 2025), a specialized metabolite–plant species database maintained by our laboratory. The KNApSAcK database is one of the most comprehensive repositories of plant-derived SMs and represents an ideal resource for identifying potential natural product-based therapeutics [21,22].

To enable computational analysis and structure-based virtual screening, all MOL-formatted structures were converted into simplified molecular input line entry system (SMILES) representations using RDKit, an open-source cheminformatics toolkit widely employed in drug discovery pipelines [57]. SMILES notation provides a compact, machine-readable format that is essential for high-throughput computational screening and molecular similarity calculations [58]. During the conversion process, 141 metabolites failed to generate valid SMILES strings because of structural inconsistencies, incomplete stereochemical information, or parsing errors in the original MOL files. After excluding these structures, the remaining 64,025 metabolites with valid SMILES representations were used for subsequent BindingDB querying and target-mapping analyses (Supplementary Materials S1, Table S1).

### 4.2. BindingDB Target Identification and HIV-1 Protein Extraction

The 64,025 validated SMILES structures were queried against BindingDB (https://www.bindingdb.org/), a public repository of experimentally determined protein–ligand binding affinities [23,24]. An automated web-scraping approach was used to retrieve binding information for each metabolite, including target protein names, species, BindingDB identifiers, and links to PDB structures. Each query used BindingDB’s SMILES similarity search at a similarity threshold of 0.8, which returns BindingDB molecules (BMs) structurally similar to the query metabolite together with each BM’s own experimentally reported target(s). The experimental affinity data therefore describes the matched BM, not the KNApSAcK metabolite itself: a metabolite is assigned to a target because it is structurally similar to a BM with confirmed activity against that target, not because the metabolite has itself been experimentally tested. This SM-to-BM-to-target chain is a standard similarity-based target-prediction strategy, but it yields a predicted target association for the metabolite, and this distinction should be kept in mind wherever these associations are used later in this study to justify a compound’s inclusion in a given target cluster. Because a single metabolite could return multiple similarity-matched targets in one BindingDB query, comma-separated multi-value fields were first exploded so that each compound–target–BindingDB-molecule (BM) association occupied its own row; rows with missing values or explicitly reporting no similarity match were then removed, and exact-duplicate rows introduced by the explosion step were dropped to yield a non-redundant compound–target association list. The initial search yielded 9756 unique metabolites linked to 7935 BMs, encompassing multiple target proteins across 234 species.

To focus on HIV-1 therapeutic targets, entries containing “HIV-1” or “Human immunodeficiency virus type 1” in the species field were selected, with emphasis on integrase, protease, reverse transcriptase, and glycoprotein targets [59,60]. No glycoprotein-binding metabolites were identified. The refined dataset (Supplementary Materials S1, Table S2) comprised 801 unique metabolites linked to 326 BMs across three HIV-1 enzymes: integrase (505 interactions), reverse transcriptase (440 interactions), and protease (169 interactions), indicating that a single metabolite could be associated with more than one binding molecule.

The PDB links associated with each interaction were accessed through the RCSB PDB (https://www.rcsb.org/) to extract the corresponding protein structure codes (Supplementary Materials S1, Table S3). This process yielded 859 unique HIV-1 protein codes, for which FASTA sequences were retrieved for subsequent clustering analysis. The metabolite-to-protein-code mappings were preserved for downstream correlation between binding profiles and cluster assignments.

### 4.3. HIV-1 Protein Sequence Clustering

FASTA sequences for the 859 unique HIV-1 protein codes were retrieved from the RCSB PDB (https://www.rcsb.org/) [25]. This dataset was filtered to retain only sequences corresponding to HIV-1 protease, reverse transcriptase, or integrase, resulting in 295 sequences for subsequent analyses (Supplementary Materials S1, Table S4). Pairwise sequence similarity was assessed using global alignment implemented in the Biopython pairwise2.align.globalxx module [39]. All-versus-all comparisons generated 43,365 pairwise alignments, calculated as

(1) 2952=295×2942=43,365.

The sequence identity was calculated as the percentage of matched residues relative to the total alignment length, including gaps. Protein–protein relationships with sequence identity ≥50% were retained to construct a weighted similarity network (Supplementary Materials S1, Table S5), in which nodes represented HIV-1 proteins and edges represented sequence identity scores [13,26,61]. Of the 295 HIV-1 protein chains subjected to alignment, 294 were assigned to one of the three clusters (135 integrase, 117 protease, 42 reverse transcriptase); the remaining chain, a 106-residue C-terminal integrase fragment included in one cryo-EM intasome structure for construct stability, did not reach 50% identity with any other chain (maximum 41.98%, against full-length integrase sequences) and was therefore left as an unclustered singleton. This threshold was chosen as a conservative cutoff within the range commonly used to distinguish confidently homologous protein regions from more divergent relationships, balancing sensitivity to genuine redundancy among HIV-1 enzyme variants against the risk of erroneously merging structures from different enzyme classes. This choice is supported directly by the pairwise alignment data underlying the similarity network (Supplementary Materials S1, Table S5): among the 16,692 same-cluster comparisons, sequence identity was high (median 96.0%, mean 89.8%), with only 2.97% of pairs falling below the 50% cutoff; among the 26,379 different-cluster comparisons, identity was low (median 19.5%, mean 18.5%), with none reaching 50% or above. The 50% threshold therefore falls within the gap separating the bulk of the same-cluster and different-cluster identity distributions, rather than being an arbitrarily chosen round number.

The similarity network was clustered using DPClusSBO1.2 [26,27,28], a graph-based clustering tool that implements density-based algorithms and was originally developed for detecting protein complexes in large interaction networks. The clustering analysis identified distinct HIV-1 protein groups based on sequence conservation. Cluster identifiers were appended to the BindingDB dataset by matching protein codes from the DPClusSBO output with those associated with each metabolite. This procedure enabled the mapping of 801 SMs to specific HIV-1 protein clusters (Supplementary Materials S1, Table S6). The cluster-annotated dataset served as the basis for subsequent molecular docking, with representative protein structures selected from each cluster and docked against their associated metabolites.

### 4.4. Molecular Docking of Secondary Metabolites to HIV-1 Protein Clusters

Proteins within the same DPClusSBO-derived cluster exhibit high sequence similarity and comparable structural and functional characteristics. Therefore, a representative PDB structure was selected for each cluster to serve as the docking target. The representative structures were refined using PyMOL v3.1.0.4 (https://pymol.org/) by removing crystallographic water molecules, ions, cofactors, and native ligands, retaining only the polymer protein chains as deposited; no missing-residue remodeling or loop-building step was performed, so any unresolved regions in the original crystallographic/cryo-EM structures remain absent in the docking receptors. Hydrogen atoms were added, and protonation states were assigned at physiological pH using LePro (https://www.lephar.com/).

Binding pockets were identified using Fpocket [32], a computational tool for detecting and characterizing protein cavities. The highest-ranking pocket score was used to define the docking-grid center and dimensions for each representative structure, computed as the pocket’s bounding box extended by 5 Å in each direction. For the main screening, this yielded grid centers of (−8.14,15.93,27.69) for 1MUI (protease) and (51.88,−27.33,37.51) for 6ELI (reverse transcriptase); for 6VDK (integrase), the fpocket-derived pocket used in the original screening was later found to be mislocated (Section 2.3) and was replaced by a grid centered directly on the native dolutegravir-binding site at (201.37,195.28,165.57), extended by the same 5 Å padding, for the corrected cluster-1 redocking; this corrected receptor also had the four crystallographic Mg^2+^ ions restored, as validated in the following subsection, and the entire cluster-1 library (285 SMs plus seven reference drugs) was subsequently redocked against this Mg^2+^-restored, re-centered receptor (Section 2.3), so all integrase docking results reported elsewhere in this study, including the MG301/MG302 interaction contacts described in Section 2, reflect this ion-restored receptor rather than the ion-free receptor used in the original, uncorrected screening. All coordinates are in the PDB structures’ native Cartesian frame (Å). SMs associated with each cluster were prepared in MOL2 format for the docking simulations.

Molecular docking was performed using SMINA [30,31] (build of 15 October 2019, based on AutoDock Vina 1.1.2), an AutoDock Vina derivative optimized for high-throughput screening. The docking parameters were set to exhaustiveness = 8 and num_modes = 10 to balance computational efficiency and conformational sampling. For each ligand–protein pair, the binding pose with the lowest predicted docking score was retained as the optimal conformation. The 2D and 3D interaction profile were also generated using BIOVIA Discovery Studio Visualizer v25.1.0.24284 for post-docking result analysis and visualization. Throughout this manuscript, docking score refers strictly to this SMINA output, and predicted interaction refers to residue-level contacts inferred from a docking pose; neither term is treated as equivalent to experimentally measured binding affinity, inhibitory activity, or antiviral efficacy, and only docking score and predicted interaction are addressed by the computational analyses performed in this study.

### 4.5. Docking Protocol Validation: Procedure

The docking protocol was validated by redocking each representative structure’s own co-crystallized native ligand into its binding site and comparing the resulting pose to the crystallographic coordinates. For each of 6VDK, 1MUI, and 6ELI, the native ligand (dolutegravir, lopinavir, and rilpivirine, respectively) was extracted from the original, un-stripped PDB coordinate file using PyMOL; for 6VDK, the four crystallographic Mg^2+^ ions were additionally extracted and merged back into the prepared receptor, since the main receptor-preparation protocol above retains only protein chains. Extracted ligands were converted to MOL2 format with explicit hydrogens using Open Babel, following the same conversion procedure used for the screening library. A validation-specific docking grid was defined by centering the SMINA search box directly on the native ligand’s own coordinates (±5 Å padding), independent of the fpocket-derived grid used for the main screening, and redocking was performed with the same SMINA parameters (exhaustiveness = 8, num_modes = 10). Pose accuracy was quantified as the heavy-atom RMSD between the top-scoring redocked pose and the crystallographic ligand, computed with OpenBabel’s OBAlign, which performs a symmetry-corrected structural superposition and does not depend on a specific input atom ordering. As a secondary diagnostic, the native-ligand-centered grid was compared against the fpocket-derived grid actually used in the main screening (Section 4.4) by computing the Euclidean distance between the two box centers, to check whether the pocket selected for large-scale screening corresponded to the biologically relevant binding site.

### 4.6. Retrospective Enrichment Validation Using DUD-E Actives and Decoys: Procedure

The validation above addresses pose accuracy, whether the top-scoring docked pose reproduces the crystallographic binding mode. It does not by itself establish whether the docking score has any discriminative validity, that is, whether it reliably scores experimentally confirmed active compounds more favorably than compounds with no reported activity. To address this second, distinct question, a retrospective enrichment analysis was performed using the Directory of Useful Decoys, Enhanced (DUD-E) [62], which provides curated sets of experimentally confirmed active compounds together with property-matched decoys (molecules with similar physicochemical properties but no reported activity, included specifically to prevent a scoring function from discriminating actives from decoys on trivial grounds such as molecular size) for named protein targets, including HIV-1 integrase (HIVINT), protease (HIVPR), and reverse transcriptase (HIVRT).

The full DUD-E sets for these three targets (HIVINT: 211 actives, 6756 decoys; HIVPR: 1395 actives, 36,278 decoys; HIVRT: 639 actives, 19,134 decoys; 64,413 compounds in total) are comparable in scale to the entire KNApSAcK screening campaign that is this study’s primary contribution, so docking the full sets was judged disproportionate for a supplementary validation check. For each target, actives and decoys were therefore subsampled deterministically (fixed random seed) to a maximum of 100 actives, with decoys sampled at a 10:1 ratio to the retained actives (up to 1000 decoys per target). All subsampled compounds were docked using SMINA with the identical parameters, grid, and representative structure already used for the corresponding target in the main screening (the Mg^2+^-restored, corrected 6VDK receptor for integrase; the unmodified 1MUI and 6ELI receptors for protease and reverse transcriptase), so that the enrichment result reflects the same protocol evaluated elsewhere in this study rather than a separately tuned setup. Compounds whose docked pose could not be sanitized for scoring (a small fraction, attributable to unusual valence states in a minority of the source structures) were excluded; this yielded final sample sizes of 98 actives and 959 decoys for HIVINT, 100 actives and 950 decoys for HIVPR, and 96 actives and 928 decoys for HIVRT.

Discriminative validity was quantified per target using the area under the receiver operating characteristic curve (ROC-AUC), treating actives as the positive class and ranking compounds by docking score (most favorable first), and the enrichment factor (EF) at the top 1%, 5%, and 10% of the ranked list, defined as the proportion of actives observed within that top fraction divided by the proportion expected under random ranking. An AUC of 0.5 and an EF of 1 both correspond to performance indistinguishable from random ranking.

### 4.7. ADME Property Prediction and Compound Prioritization

Although molecular docking provides insights into predicted binding, ADME properties are critical for assessing drug-likeness and oral bioavailability [13,33]. The SMILES representations of the docked SMs were subjected to SwissADME analysis (http://www.swissadme.ch/, accessed 21 May 2026) [33] for comprehensive pharmacokinetic profiling. SwissADME predicts key physicochemical descriptors, lipophilicity, water solubility, pharmacokinetic parameters, and drug-like properties based on established rules and computational models [33].

Owing to the technical limitations of SwissADME, which accepts SMILES inputs of up to 200 characters, certain large SMs could not be analyzed and were therefore excluded from ADME-based screening. Final candidates were selected using an explicit, reproducible ranked-screening procedure applied independently within each cluster: docked SMs were first ranked by docking score (most favorable first); starting from the top of this ranking, each compound was evaluated in turn, skipping any compound whose SMILES representation exceeded SwissADME’s 200-character limit and any compound whose bioavailability radar profile fell outside the optimal (pink) region, until three compounds satisfying both criteria were identified. This same rank-then-screen procedure was applied uniformly to all three clusters and is the sole criterion underlying the three final candidates reported for each target in Section 2. Beyond the six bioavailability radar properties used for this initial screening, the nine final candidates were additionally profiled using SwissADME’s gastrointestinal absorption, blood–brain barrier permeation, P-glycoprotein substrate, cytochrome P450 (CYP1A2, CYP2C19, CYP2C9, CYP2D6, CYP3A4) inhibition, Ghose/Veber/Egan/Muegge drug-likeness rule sets, and PAINS/Brenk structural-alert predictions (Table 6).

Toxicity was not evaluated by SwissADME and was therefore assessed separately using ProTox 3.0 (https://tox.charite.de/protox3/, accessed 29 July 2026) [42], a structure–activity-based toxicity prediction server. Each of the nine final candidates was submitted by canonical SMILES and screened for predicted acute oral toxicity (LD50, GHS classification) together with five endpoints: hepatotoxicity, hERG-related cardiotoxicity, carcinogenicity, Ames-related mutagenicity, and cytotoxicity. For two compounds (C00017264 and C00001695), direct SMILES submission failed ProTox’s structure-conversion step; these two were instead submitted via PubChem-name-matched search using their identified common names (18-Deoxycytochalasin H and Brucine, respectively), which the server converts to structure internally.

### 4.8. Comparative Validation Using FDA-Approved and Clinical HIV-1 Drugs

To contextualize the docking performance of the prioritized SMs, a comparative validation analysis was conducted using FDA-approved and clinical-stage HIV-1 inhibitors retrieved from the ChEMBL database (accessed 20 May 2026) [63]. Reference compounds were retrieved for each HIV-1 enzyme class using the ChEMBL target identifiers for integrase (CHEMBL3471), protease (CHEMBL243), and reverse transcriptase (CHEMBL247). Only approved drugs and clinical candidates were included in the comparative dataset (Supplementary Materials S2, Table S7).

FDA-approved and clinical-stage HIV-1 inhibitors underwent the same ligand-preparation protocol as the SMs, including structural conversion, protonation, and energy minimization. These reference compounds were docked using identical SMINA parameters against the same representative protein structures for each cluster. This ensured a direct and controlled comparison between the predicted docking scores of the natural product candidates and those of established clinical therapeutics. The docking scores of the FDA-approved inhibitors served as benchmarks for assessing whether the ADME-prioritized SMs exhibited docking scores comparable to or more favorable than those of current HIV-1 therapeutics.

## 5. Conclusions

This study presents a cluster-guided, redundancy-aware virtual screening framework for the large-scale prioritization of natural product candidates targeting HIV-1. By organizing structurally related HIV-1 protein variants through sequence-based clustering prior to docking, the framework efficiently reduced redundancy in target selection while preserving coverage of the three major viral enzyme classes (integrase, protease, and reverse transcriptase). However, the medoid-sensitivity analysis (Table 10) showed that two of the three representative structures used were themselves atypical members of their own clusters and that protease results in particular are sensitive to this choice, so representative-structure selection remains an important caveat on how far single-structure screening within each cluster can be generalized. A retrospective enrichment analysis using DUD-E actives and decoys further showed that the docking score carries genuine discriminative validity, beyond internal consistency alone, for integrase and protease (ROC-AUC 0.845 and 0.783), directly supporting the prioritization approach used for these two targets; the same analysis showed no such validity for reverse transcriptase (ROC-AUC 0.529), consistent with the comparatively poor pose-reproduction accuracy already documented for that target, and this caveat should be weighed when interpreting reverse-transcriptase candidates specifically. This strategy enabled the systematic processing of more than 64,000 KNApSAcK metabolites through SMILES conversion and BindingDB-based target mapping, from which 801 unique compounds mapped to HIV-1 integrase, protease, or reverse transcriptase and were subsequently docked against the corresponding representative structures, followed by ADME-based prioritization to identify compounds with favorable drug-like properties.

The results showed that several natural product metabolites achieved competitive or, in some cases, more favorable predicted docking scores compared with FDA-approved HIV-1 inhibitors, particularly for reverse transcriptase. After integrating docking performance with ADME-based prioritization, C00002145 (Camptothecin), C00017264 (18-Deoxycytochalasin H; L 696474), and C00003719 (Limonin) emerged as the top-ranked candidates for integrase, protease, and reverse transcriptase, respectively. In addition, the drug-like properties of two other compounds were evaluated for each of the three target classes, for a total of nine computationally prioritized candidates. ProTox 3.0 toxicity screening of these nine candidates (Table 7) revealed a cluster-specific pattern of concern: all three integrase candidates, the camptothecin family, were predicted cytotoxic with high confidence, consistent with camptothecin’s established mechanism as a cytotoxic topoisomerase I poison, and C00001695 (Brucine) additionally showed the lowest predicted acute oral toxicity threshold among the reverse-transcriptase candidates (second-lowest of all nine, after the three integrase compounds) and a predicted-active carcinogenicity flag. These findings do not necessarily rule out the affected candidates, since predicted cytotoxicity and antiviral activity operate through distinct mechanisms and structural optimization could in principle decouple them, but they meaningfully temper the integrase cluster’s otherwise favorable profile and should be weighed alongside docking score when selecting candidates for experimental follow-up. All nine compounds should be regarded as computationally prioritized hypotheses requiring experimental validation of both antiviral activity and safety, not as validated therapeutics.

Beyond the specific application to HIV-1, the main contribution of this study lies in demonstrating how sequence-similarity-based target organization can improve the scalability, interpretability, and biological coherence of large-scale virtual screening workflows. This study also completes the reverse, compound-first direction of a bidirectional BindingDB-mediated screening strategy: whereas our previous work began from a single viral protein sequence to retrieve candidate binding molecules and, from these, prioritize small-molecule inhibitors against SARS-CoV-2 [13], the present study begins from a large compound library and uses BindingDB associations to identify the relevant protein targets. Together, the two studies indicate that the same sequence-similarity-network-guided approach generalizes across both screening directions. The proposed framework provides a transferable strategy for redundancy-aware screening in systems characterized by multiple related target variants and large compound libraries. Nevertheless, the current study is limited by its reliance on docking-based prioritization and representative target structures, which cannot fully capture protein flexibility, resistance-associated variation, or true inhibitory activity. Future studies should focus on integrating experimental validation, resistance-aware target modeling, and higher-resolution structural refinement to evaluate and strengthen the predictive utility of this framework.

## Figures and Tables

**Table 1:** Summary of the HIV-1 protein sequence clustering results.

Cluster ID	Enzyme Class	Representative PDB	Number of Proteins
1	Integrase	6VDK	135
2	Protease	1MUI	117
3	Reverse Transcriptase	6ELI	42

**Table 2:** Distribution of SMs across the HIV-1 protein clusters.

Cluster ID	Enzyme Class	Representative PDB	Number of Unique SMs
1	Integrase	6VDK	285
2	Protease	1MUI	124
3	Reverse Transcriptase	6ELI	410

**Table 3:** Docking protocol validation: redocking RMSD (default and follow-up correction attempts) and grid-placement comparison for the three representative structures.

PDB (Enzyme)	Native Ligand	Default RMSD: Top-Pose (Best-of-10) (Å)	Ex. = 16 RMSD (Å)	Flexible-Residue RMSD (Å)	Native- vs. Screening-Grid Distance (Å)
6VDK (Integrase)	Dolutegravir	6.44 (6.32)	N/A ^2^	N/A ^2^	28.78 ^1^
1MUI (Protease)	Lopinavir	5.91 (5.66)	5.81	5.82 ^3^	1.10
6ELI (Reverse Transcriptase)	Rilpivirine	5.61 (4.86)	4.98	4.97 ^3^	3.19

^1^ Distance from the native ligand to the original, uncorrected fpocket-derived grid diagnosed by this validation step. This grid was subsequently replaced with one centered directly on the native dolutegravir coordinates (distance to native ligand = 0 Å by construction), and the full cluster-1 compound library was redocked against the corrected grid before any integrase results reported elsewhere in this study were generated; see the main text below and Section 4.4. This 28.78 Å value does not apply to the corrected protocol or to any reported result. ^2^ Not applicable: the 6VDK redocking failure was traced to a grid-placement error rather than to sampling depth or induced-fit behavior, and was corrected by re-centering the grid (see main text) rather than by the exhaustiveness or flexible-residue diagnostics developed for 1MUI and 6ELI. ^3^ Flexible side chains at ILE50 of both protease monomers (1MUI) and at TYR181, TYR188, and TRP229 of the NNRTI pocket (6ELI), docked at the default exhaustiveness using SMINA’s –flexres mode; see the main text for rationale.

**Table 4:** Retrospective enrichment validation using DUD-E actives and property-matched decoys, docked under the identical protocol, parameters, and representative structures used for the main screening of each target.

PDB (Enzyme)	N (Actives/Decoys)	ROC-AUC	EF 1%	EF 5%	EF 10%
6VDK (Integrase)	98/959	0.845	5.88	5.49	4.48
1MUI (Protease)	100/950	0.783	6.30	4.24	3.80
6ELI (Reverse Transcriptase)	96/928	0.529	2.13	1.05	1.25

**Table 5:** Top five SMs per cluster ranked by docking scores. Cluster 1 (integrase) values reflect the corrected docking protocol described in Section 2.3 (grid centered on the validated dolutegravir-binding site rather than the original fpocket-derived pocket).

Cluster ID	Enzyme Class	Representative PDB	SMs (CID)	Docking Score (kcal/mol)
1	Integrase	6VDK	C00002829	−18.91
			C00016684	−14.98
			C00006095	−14.46
			C00015144	−14.42
			C00006268	−14.31
2	Protease	1MUI	C00009310	−26.54
			C00009309	−24.28
			C00009311	−24.11
			C00009304	−23.75
			C00009303	−23.74
3	Reverse Transcriptase	6ELI	C00002268	−26.83
			C00001783	−26.81
			C00001781	−26.68
			C00001895	−26.60
			C00001919	−26.16

**Table 6:** Extended pharmacokinetic and drug-likeness profile of the nine final candidates.

Enzyme	CID	GI Abs.	BBB	P-gp	CYP Inhibited	Rule Violations ^1^	BAS	PAINS	Brenk	SA Score
Integrase	C00002145	High	No	Yes	1A2, 2C9, 3A4	0	0.55	0	0	3.84
Integrase	C00052100	High	No	Yes	1A2	0	0.55	0	0	3.88
Integrase	C00064112	High	No	Yes	1A2	0	0.55	0	0	3.84
Protease	C00017264	High	No	Yes	3A4	2 (Ghose)	0.55	0	1	6.64
Protease	C00048932	High	No	Yes	2D6	0	0.56	1	1	4.04
Protease	C00036880	High	No	No	2C9	0	0.56	1	1	3.81
RT	C00003719	High	No	No	None	0	0.55	0	2	6.49
RT	C00001778	High	**Yes** ^2^	Yes	None	2 (Ghose)	0.55	1	0	4.95
RT	C00001695	High	**Yes** ^2^	No	2D6	0	0.55	0	1	5.44

^1^ Non-Lipinski rule-set violations (Ghose/Veber/Egan/Muegge); Lipinski violations were 0 for all nine compounds. GI Abs. = gastrointestinal absorption; BBB = blood–brain barrier permeant; P-gp = P-glycoprotein substrate; BAS = Bioavailability Score; SA Score = Synthetic Accessibility Score (1 = easy, 10 = difficult). ^2^ Bold denotes the two BBB-permeant candidates discussed in the main text as warranting consideration of off-target central nervous system exposure.

**Table 7:** ProTox 3.0 predicted acute oral toxicity and active toxicological endpoint(s) for the nine final candidates. All endpoints not listed under “Active Endpoint(s)” were predicted inactive for that compound.

Enzyme	SM (CID)	Predicted LD50	GHS Class ^1^	Active Endpoint(s)	Probability
Integrase	C00002145	50 mg/kg	3	Cytotoxicity	0.98
Integrase	C00052100	50 mg/kg	3	Cytotoxicity	0.96
Integrase	C00064112	50 mg/kg	3	Cytotoxicity	0.96
Protease	C00017264	4000 mg/kg	5	Hepatotoxicity	0.60
Protease	C00048932	287 mg/kg	3	Cardiotoxicity	0.61
Protease	C00036880	287 mg/kg	3	Cardiotoxicity	0.56
RT	C00003719	244 mg/kg	3	Cardiotoxicity	0.53
RT	C00001778	760 mg/kg	4	None	–
RT	C00001695	150 mg/kg	3	Carcinogenicity	0.53

^1^ GHS acute oral toxicity classification: Class 3 = toxic if swallowed (50 < LD50 ≤ 300 mg/kg); Class 4 = harmful if swallowed (300 < LD50 ≤ 2000 mg/kg); Class 5 = may be harmful if swallowed (2000 < LD50 ≤ 5000 mg/kg).

**Table 8:** Statistical comparison summary of docking scores (kcal/mol) between ADME-prioritized SMs (SM) and FDA-approved HIV-1 inhibitors (AD) across the three protein clusters.

Cluster	SM Mean ± SD (*n*)	AD Mean ± SD (*n*)	Mann–Whitney *U* (*p*-Value)	Cohen’s *d*
Cluster 1 (6VDK)	−7.29 ± 2.16 (n=12)	−8.03 ± 0.43 (n=7)	56.0 (0.261)	0.480
Cluster 2 (1MUI)	−11.96±0.49 (n=3)	−10.55±1.07 (n=5)	2.0 (0.143)	−1.692
Cluster 3 (6ELI)	−13.78±2.31 (n=144)	−10.99±1.09 (n=12)	191.0 (<0.001)	−1.540

Significance was assessed using the Mann–Whitney *U* test (two-sided *p*-values). Effect size was measured using Cohen’s *d*, where |d|<0.2 was negligible, 0.2–0.8 was small to medium, and |d|>0.8 was large. For Cluster 3, the approved-drug comparator (AD) is restricted to the 12 non-nucleoside reverse transcriptase inhibitors (NNRTIs) among the 31 originally retrieved reference compounds; the remaining 19 nucleoside/nucleotide reverse-transcriptase inhibitors (NRTIs/NtRTIs) act at a mechanistically distinct site and are reported separately in the text rather than pooled into this comparison (see the main text for rationale).

**Table 9:** Compound-level comparison: top-ranked ADME-prioritized SM per cluster versus its target’s own co-crystallized reference inhibitor, redocked into the native site.

Enzyme	Top SM (CID)	SM Score	Co-Crystallized Ligand	Ligand Score ^1^	Difference
Integrase	C00002145	−12.70	Dolutegravir (DLU)	−7.54	−5.16
Protease	C00017264	−12.63	Lopinavir (AB1)	−11.04	−1.59
Reverse Transcriptase	C00003719	−20.95	Rilpivirine (T27)	−8.84	−12.11

^1^ Top-scoring pose from redocking the co-crystallized ligand into its own native site (Table 3), not the originally deposited crystallographic pose. All values in kcal/mol; more negative indicates a more favorable predicted docking score.

**Table 10:** Medoid sensitivity check: ADME-prioritized final candidates redocked against each cluster’s medoid structure, compared with the original representative structure.

Enzyme	SM (CID)	Original PDB	Original Score	Medoid PDB	Medoid Score
Integrase	C00002145	6VDK	−12.70	4CJR	−12.24
	C00052100		−9.75		−10.90
	C00064112		−8.83		−9.66
Protease	C00017264	1MUI	−12.63	7LE4	−8.86
	C00048932		−11.79		−9.93
	C00036880		−11.46		−9.11

## Data Availability

Scripts and notebooks are available at https://github.com/xevworks/knapsack-hiv-publish (accessed on 9 September 2026). The full dataset was deposited in Zenodo (DOI: 10.5281/zenodo.21681014). Data supporting the findings of this study are also available in the online Supplementary Materials. Further inquiries can be directed to the corresponding author.

## Supplementary Materials

The following supporting information can be downloaded at https://www.mdpi.com/article/10.3390/ph19101534/s1: Supplementary Materials S1 (Tables S1–S6): KNApSAcK secondary metabolite SMILES conversion, BindingDB target-mapping, RCSB PDB code extraction, protein sequence, similarity-network, and cluster-assignment data; Supplementary Materials S2 (Figures S1–S3 and Tables S7–S11): complete network visualization of the structural clustering of HIV-1 enzymes, SwissADME bioavailability radar profiles of top-docking-ranked secondary metabolites for Clusters 1 and 3, the ChEMBL reference-compound list, and per-cluster docking-interaction tables; Supplementary Materials S3 (Tables S12–S15): docking-score distributions and statistical-comparison descriptive statistics for all three clusters.

## Abbreviations

The following abbreviations are used in this manuscript:

AD	Approved Drug
ADME	Absorption, Distribution, Metabolism, and Excretion
ART	Antiretroviral Therapy
BAS	Bioavailability Score
BM	Binding Molecule
CID	Compound Identifier
Cryo-EM	Cryo-Electron Microscopy
FDA	Food and Drug Administration
FLEX	Flexibility
HIV-1	Human Immunodeficiency Virus Type 1
INSATU	Saturation
INSOLU	Solubility
INSTI	Integrase Strand-Transfer Inhibitors
IQR	Interquartile Range
KDE	Kernel Density Estimates
LEDGF	Lens Epithelium-Derived Growth Factor
LIPO	Lipophilicity
MD	Molecular Dynamics
MW	Molecular Weight
NNRTI	Non-Nucleoside Reverse-Transcriptase Inhibitors
PDB	Protein Data Bank
PDBJ	Protein Data Bank Japan
POLAR	Polarity
RT	Reverse Transcriptase
SAR	Structure–Activity Relationship
SD	Standard Deviation
SIZE	Molecular Size
SM	Secondary Metabolite
SMILES	Simplified Molecular Input Line Entry System
TPSA	Topological Polar Surface Area
