# Supplementary material for: A Cluster-Guided Screening Framework for Prioritizing Natural Product Candidates Against HIV-1 from the KNApSAcK Database
Source: Pharmaceuticals (Basel). 2026 Sep 27;19(10):1534. doi: 10.3390/ph19101534 (PMC13708532; doi:10.3390/ph19101534)
Supplement: Supplementary file 1 [file pharmaceuticals-19-01534-s001.zip › Supplementary Materials S2.pdf]

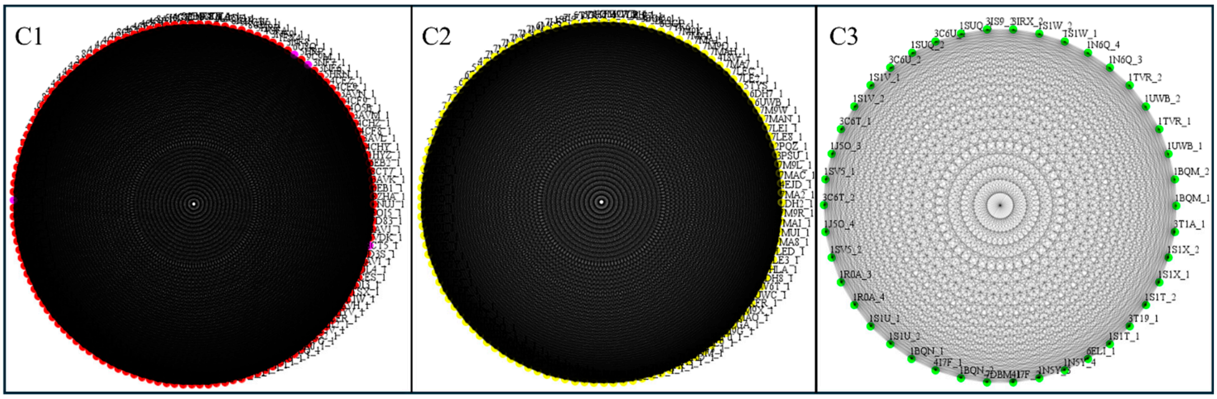

Figure S1. Complete network visualization of the structural clustering of HIV-1 enzymes.

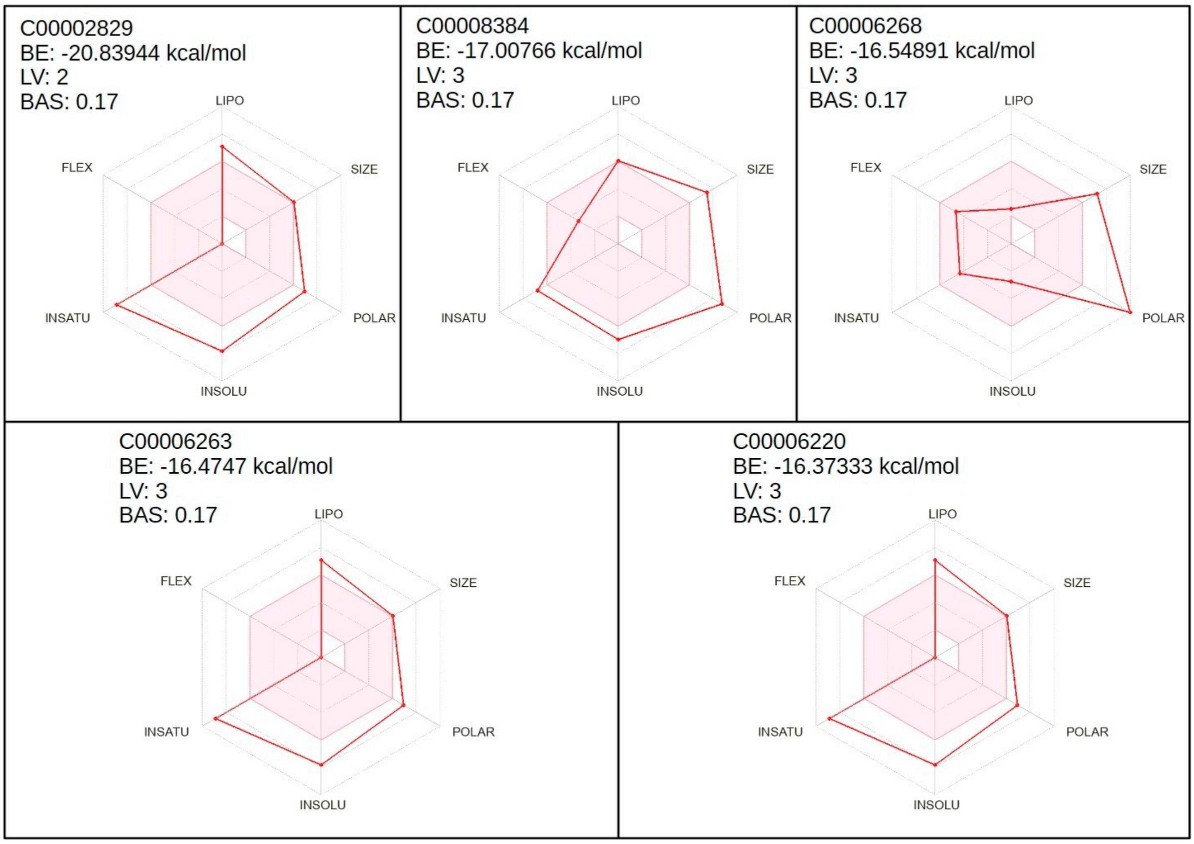

Figure S2. SwissADME bioavailability radar profiles of top-docking-ranked SMs for Cluster 1 (integrase).

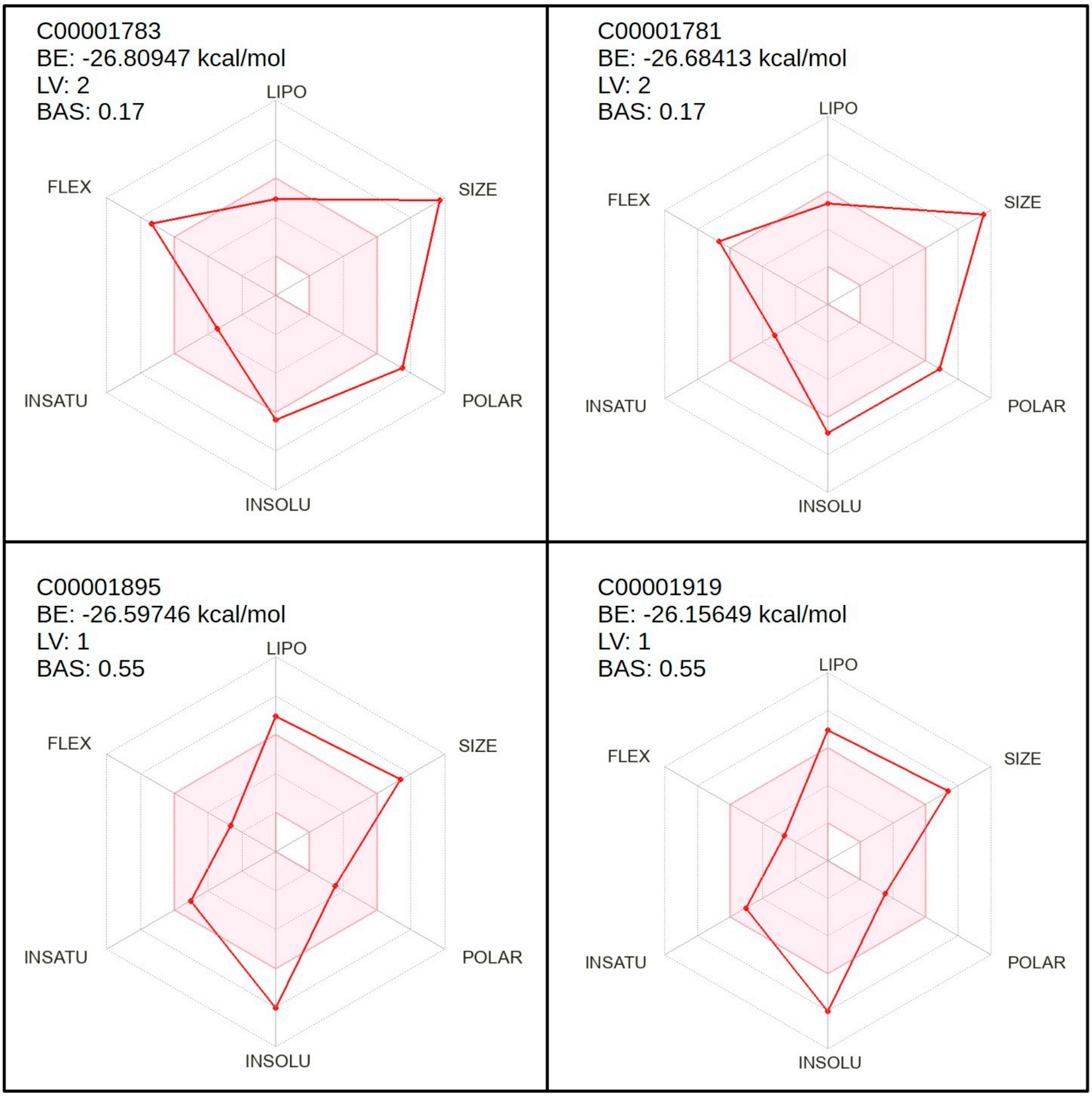

Figure S3. SwissADME bioavailability radar profiles of docking-ranked SMs for Cluster 3 (HIV-1 reverse transcriptase).

Table S7. FDA-approved drugs for comparative analysis.

Cluster	Class	Chembl ID	FDA Drug	Mechanism
1	Integrase	CHEMBL3471	DOLUTEGRAVIR	INSTI
			BICTEGRAVIR	
			CABOTEGRAVIR	
			RALTEGRAVIR	
			ELVITEGRAVIR	
			GSK364735	
			S1360	
2	Protease	CHEMBL243	SAQUINAVIR	PI

			INDINAVIR	
			NELFINAVIR	
			RITONAVIR	
			DARUNAVIR	
3	Reverse Transcriptase	CHEMBL247	BILR355	NNRTI
			RILPIVIRINE	
			DELAVIRDINE	
			EFAVIRENZ	
			DORAVIRINE	
			ETRAVIRINE	
			NEVIRAPINE	
			ATEVIRDINE	
			DAPIVIRINE	
			CAPRAVIRINE	
			EMIVIRINE	
			LERSIVIRINE	
			ROVAFOVIR	NRTI
			TENOFOVIR EXALIDEX	
			ABACAVIR	
			ISLATRAVIR	
			TENOFOVIR ALAFENAMIDE	
			TENOFOVIR DISOPROXIL	
			AMDOXOVIR	
			CENSAVUDINE	
			ZIDOVUDINE	
			AZVUDINE	
			STAVUDINE	
			DIDANOSINE	
			TENOFOVIR	
			ELVUCITABINE	
			DEXELVUCITABINE	
			ZALCITABINE	
			APRICITABINE	
			EMTRICITABINE	
			LAMIVUDINE	

Table S8. HIV-1 Protein Sequence Clustering Results

Cluster ID	Size	Edge	Density	Enzyme Class	Proteins
1	135	8549	0.95	Integrase	6U8Q_1, 6VDK_1, 8W2R_1, 9C9M_1, 1EX4_1, 1K6Y_1, 6VRG_1, 8USY_1, 8V0Z_1, 8V9C_1, 8W09_1, 8W34_1, 9BW9_2, 9C29_1, 1HYV_1, 1HYZ_1, 3AV9_1, 3AVA_1, 3AVB_1, 3AVC_1, 3AVG_1, 3AVH_1, 3AVI_1, 3AVJ_1, 3AVK_1, 3AVL_1, 3AVM_1, 3AVN_1, 3LPT_1, 3LPU_1, 3NF6_1, 3NF7_1, 3NF8_1, 3NF9_1, 3NFA_1, 4AH9_1, 4AHR_1, 4AHS_1, 4AHT_1, 4AHU_1, 4AHV_1, 4CE9_1, 4CEA_1, 4CEB_1, 4CEC_1, 4CED_1, 4CEE_1, 4CEF_1, 4CEO_1, 4CEQ_1, 4CER_1, 4CES_1, 4CEZ_1, 4CF0_1, 4CF1_1, 4CF2_1, 4CF8_1, 4CF9_1, 4CFA_1, 4CFB_1,

Cluster ID	Size	Edge	Density	Enzyme Class	Proteins
					4CFC_1, 4CFD_1, 4CGD_1, 4CGF_1, 4CGG_1, 4CGH_1, 4CGI_1, 4CGJ_1, 4CHN_1, 4CHO_1, 4CHP_1, 4CHQ_1, 4CHY_1, 4CHZ_1, 4CIE_1, 4CIF_1, 4CIG_1, 4CJ3_1, 4CJ5_1, 4CJE_1, 4CJF_1, 4CJK_1, 4CJL_1, 4CJP_1, 4CJQ_1, 4CJR_1, 4CJS_1, 4CJT_1, 4CJU_1, 4CJV_1, 4CJW_1, 4CK1_1, 4CK2_1, 4CK3_1, 4LH4_1, 4LH5_1, 4O0J_1, 4O55_1, 4O5B_1, 4OVL_1, 4TSX_1, 5HRP_1, 5KGW_1, 5KGX_1, 5OI2_1, 5OI3_1, 5OI5_1, 6EB1_1, 6EB2_1, 6JCF_1, 6JCG_1, 6L0C_1, 6NUJ_1, 7D83_1, 7KE0_1, 8BV2_1, 8CT5_1, 8CT7_1, 8D3S_1, 8S9Q_1, 8T52_1, 8ZH4_1, 8ZHA_1, 8A1P_1, 8A1Q_1, 8BUV_1, 8CBR_1, 8CBS_1, 8CBT_1, 8CBU_1, 8CBV_1, 5HRN_1, 5HRR_1, 5HRS_1, 5JL4_1
2	117	6786	1	Protease	1MUI_1, 1SP5_1, 2BB9_1, 2BBB_1, 2HB4_1, 2PC0_1, 2PQZ_1, 2WKZ_1, 2ZGA_1, 3BGB_1, 3BGC_1, 3BHE_1, 3CKT_1, 3KF0_1, 3KFN_1, 3KFP_1, 3KFR_1, 3KFS_1, 3PSU_1, 4E43_1, 4EJD_1, 4EJK_1, 4HLA_1, 4I8W_1, 4I8Z_1, 4LL3_1, 4TVH_1, 5IVQ_1, 5IVR_1, 5IVS_1, 5TYR_1, 5TYS_1, 5VCK_1, 6D0D_1, 6D0E_1, 6DGX_1, 6DGY_1, 6DGZ_1, 6DH2_1, 6DH6_1, 6DH7_1, 6DH8_1, 6MCR_1, 6MCS_1, 6OGP_1, 6OYD_1, 6OYR_1, 6UWB_1, 6UWC_1, 6W6T_1, 7LDY_1, 7LDZ_1, 7LE0_1, 7LE1_1, 7LE2_1, 7LE3_1, 7LE4_1, 7LE5_1, 7LE6_1, 7LE7_1, 7LE8_1, 7LE9_1, 7LEA_1, 7LEB_1, 7LEC_1, 7LED_1, 7LEE_1, 7LEF_1, 7LEG_1, 7LEH_1, 7LEI_1, 7M9G_1, 7M9H_1, 7M9I_1, 7M9J_1, 7M9K_1, 7M9L_1, 7M9M_1, 7M9N_1, 7M9O_1, 7M9P_1, 7M9Q_1, 7M9R_1, 7M9S_1, 7M9T_1, 7M9U_1, 7M9V_1, 7M9W_1, 7M9X_1, 7M9Z_1, 7MA0_1, 7MA1_1, 7MA2_1, 7MA3_1, 7MA4_1, 7MA5_1, 7MA6_1, 7MA7_1, 7MA8_1, 7MA9_1, 7MAA_1, 7MAB_1, 7MAC_1, 7MAD_1, 7MAE_1, 7MAF_1, 7MAG_1, 7MAH_1, 7MAI_1, 7MAJ_1, 7MAK_1, 7MAL_1, 7MAN_1, 7MAO_1, 7WBS_1, 7WCQ_1, 7YF6_1
3	42	861	1	Reverse Transcriptase	1BQM_2, 1BQM_1, 1BQN_2, 1BQN_1, 1J5O_4, 1J5O_3, 1N5Y_4, 1N5Y_3, 1N6Q_3, 1N6Q_4, 1R0A_4, 1R0A_3, 1S1T_2, 1S1T_1, 1S1U_2, 1S1U_1, 1S1V_2, 1S1V_1, 1S1W_2, 1S1W_1, 1S1X_1, 1S1X_2, 1SUQ_2, 1SUQ_1, 1SV5_2, 1SV5_1, 1TVR_2, 1TVR_1, 1UWB_2, 1UWB_1, 3C6T_2, 3C6T_1, 3C6U_2, 3C6U_1, 3IRX_2, 3IS9_2, 3T19_1, 3T1A_1, 4I7F_2, 4I7F_1, 6ELI_1, 7DBM_1

Table S9. Comprehensive result of docking and binding interaction analysis of top three secondary metabolites for cluster 1

Ligand	Docking Score (kcal/mol)	Residue in Contact	Bond Category	Interaction Type	Distance (Å)
C00002145	-12.6994	ASN144	Hydrogen Bond	Conventional Hydrogen Bond	2.94598
		PRO142	Hydrogen Bond	Conventional Hydrogen Bond	2.76298
		TYR143	Hydrogen Bond	Carbon Hydrogen Bond	2.32641
		PRO145	Hydrogen Bond	Carbon Hydrogen Bond	2.94936

Ligand	Docking Score (kcal/mol)	Residue in Contact	Bond Category	Interaction Type	Distance (Å)
		PRO145	Hydrophobic	Pi-Alkyl	4.09932
		PRO145	Hydrophobic	Pi-Alkyl	3.88457
		PRO145	Hydrophobic	Pi-Alkyl	3.97007
		MG301	Electrostatic	Pi-Cation	4.00593
		ASP116	Electrostatic	Pi-Anion	3.51935
		ASP116	Electrostatic	Pi-Anion	3.56326
		ASP116	Electrostatic	Pi-Anion	4.25587
C00052100	-9.74686	ASN144	Hydrogen Bond	Conventional Hydrogen Bond	2.8855
		TYR143	Hydrogen Bond	Carbon Hydrogen Bond	2.8487
		PRO145	Hydrogen Bond	Carbon Hydrogen Bond	2.66512
		GLU152	Hydrogen Bond	Carbon Hydrogen Bond	3.54097
		MG301	Electrostatic	Pi-Cation	3.71787
		MG301	Electrostatic	Pi-Cation	4.022
		ASP116	Electrostatic	Pi-Anion	4.01509
		ASP116	Electrostatic	Pi-Anion	3.62538
		PRO145	Hydrophobic	Pi-Alkyl	4.75809
		PRO145	Hydrophobic	Pi-Alkyl	3.86876
C00064112	-8.83199	ILE141	Hydrogen Bond	Conventional Hydrogen Bond	2.17755
		MG302	Other	Metal-Acceptor	2.27561
		MG301	Electrostatic	Pi-Cation	3.07854
		ASP64	Electrostatic	Pi-Anion	4.532
		ASP116	Electrostatic	Pi-Anion	3.75772
		ASP116	Electrostatic	Pi-Anion	3.39495
		ASP116	Electrostatic	Pi-Anion	4.19878
		PRO145	Hydrophobic	Pi-Alkyl	4.50761
		PRO145	Hydrophobic	Pi-Alkyl	4.62076

Table S10. Comprehensive result of docking and binding interaction analysis of top three secondary metabolites for cluster 2

Ligand	Docking Score (kcal/mol)	Residue in Contact	Bond Category	Interaction Type	Distance (Å)
C00017264	-12.62836	ILE50	Hydrogen Bond	Conventional Hydrogen Bond	3.02465
		ALA28	Hydrophobic	Pi-Alkyl	4.80625
		VAL32	Hydrophobic	Pi-Alkyl	5.32482
		ILE84	Hydrophobic	Pi-Alkyl	4.75434
		ILE50	Hydrophobic	Pi-Alkyl	4.06163
C00048932	-11.79474	ASP29	Hydrogen Bond	Conventional Hydrogen Bond	2.48492
		ASP29	Hydrogen Bond	Conventional Hydrogen Bond	2.29531
		ASP29	Hydrogen Bond	Conventional Hydrogen Bond	2.74935
		ASP30	Hydrogen Bond	Conventional Hydrogen Bond	1.92188

		ASP30	Hydrogen Bond	Conventional Hydrogen Bond	2.2266
		ASP30	Hydrogen Bond	Conventional Hydrogen Bond	2.14479
		ASP30	Hydrogen Bond	Conventional Hydrogen Bond	2.54189
		ASP29	Hydrogen Bond	Conventional Hydrogen Bond	3.18155
		ALA28	Hydrophobic	Pi-Alkyl	4.98888
		ILE47	Hydrophobic	Pi-Alkyl	4.79915
C00036880	-11.45782	ASP30	Hydrogen Bond	Conventional Hydrogen Bond	2.16618
		ASP30	Hydrogen Bond	Conventional Hydrogen Bond	2.25247
		ASP30	Hydrogen Bond	Conventional Hydrogen Bond	2.67679
		ILE47	Hydrophobic	Pi-Alkyl	4.16887

Table S11. Comprehensive result of docking and binding interaction analysis of top three secondary metabolites for cluster 3

Ligand	Docking Score (kcal/mol)	Residue in Contact	Bond Category	Interaction Type	Distance (Å)
C00003719	-20.94702	GLU138	Hydrogen Bond	Carbon Hydrogen Bond	2.5035
		TYR181	Hydrophobic	Pi-Sigma	3.7999
		TRP229	Hydrophobic	Pi-Sigma	3.76431
		LEU100	Hydrophobic	Pi-Alkyl	5.29821
		VAL179	Hydrophobic	Pi-Alkyl	4.26106
C00001778	-19.41552	TYR181	Electrostatic	Pi-Anion	4.78038
		TYR188	Hydrophobic	Pi-Sigma	3.32023
		TYR188	Hydrophobic	Pi-Sigma	3.56492
		TRP229	Hydrophobic	Pi-Sigma	3.67254
		TYR188	Hydrophobic	Pi-Pi Stacked	3.6343
		PHE227	Hydrophobic	Pi-Pi T-shaped	5.25516
		TRP229	Hydrophobic	Pi-Pi T-shaped	4.20896
		TRP229	Hydrophobic	Pi-Pi T-shaped	5.10383
		LEU234	Hydrophobic	Pi-Alkyl	5.40519
C00001695	-19.0299	TYR188	Hydrophobic	Pi-Sigma	3.6492
		TRP229	Hydrophobic	Pi-Sigma	3.65513
		TYR188	Hydrophobic	Pi-Sigma	3.68599
		TYR188	Hydrophobic	Pi-Pi Stacked	3.69911
		PHE227	Hydrophobic	Pi-Pi T-shaped	5.33774
		TRP229	Hydrophobic	Pi-Pi T-shaped	4.44791
		LEU234	Hydrophobic	Pi-Alkyl	5.39839
